# Standards, Options and Recommendations: SOR organising committee: FNCLCC, Paris

**DOI:** 10.1038/sj.bjc.6601094

**Published:** 2003-08-15

**Executive committee**

**T Philip**, Director of SOR, paediatrician

**B Fervers**, Deputy director of SOR, medical oncologist

**P Bey**, Representant from the FNCLCC Advisory Committee, radiotherapist

**D Maigne**, General manager of the FNCLCC

**SOR specialist project**

**A Bataillard**, Coordinator of the SOR specialist project, general practitioner

**G Gory-Delabaere**, Methodologist, pharmacist

**M Haugh**, Methodologist, biochemist

**F Farsi**, Network referent, public health physician

**E Luporsi**, Associated methodologist, medical oncologist

**S Theobald**, Associated methodologist, public health physician

**L Bosquet**, Assistant methodologist

**N Fabre**, Assistant methodologist

**S Rousmans**, Assistant methodologist

**SOR SAVOIR patient project**

**S Brusco**, Methodologist

**J Carretier**, Methodologist

**V Delavigne**, Linguist

**L Leichtnam-Dugarin**, Methodologist

**Scientific information service**

**S Guillo**, Information scientist

**AG Guy**, Information technician

**Administration and editorial service**

**S Debuiche**, Head of service

**H Borges-Paninho**, Document manager

**E Esteves**, Secretary

**D Ropé**, Assistant

**Scientific Advisory Committee**

**I Durand-Zaleski** (President), Hôpital Henri Mondor, France

**J Clavier** (Vice-President), Hôpital Morvan, France

**JP Boissel**, Hôpital Neuro-Cardiologique, France

**C Chouaïd**, Hôpital St Antoine, France

**F Cluzeau**, St George's Hospital, UK

**P Durieux**, Faculté de médecine–Hôtel Dieu, France

**C Esper**, Centre National de l'Equipement Hospitalier, France

**R Gagnayre**, UFR de Bobigny, France

**A Giraud**, Hôpital Jean Verdier, France

**G Ollenschläger**, Agency for Quality in Medicine, Germany

**R Otter**, Integraal Kankercentrum Noord Nederland, The Netherlands

**R Pinkerton**, Royal Marsden Hospital, UK

**D Serin**, Clinique Ste Catherine, France

**G Viens**, Chaire Essec Santé, France

*One representative from each of the following institutions*:

AERIO (**B Besse**), CNAMTS, DGS (**MF Chedru**), INSERM (**C Moquin-Pattey**), Ligue Nationale Contre le Cancer (**H Pujol**), OECI (**G Storme, Belgium**), UNHPC (**N Albin**), FHF (**L Cals**), College of General Managers of University Hospitals (**F Grateau**), College of Presidents of University Hospital Medical Committees (**J Lansac**), France

Also a representative for patients (**S Delahaye**), and for general practitioners (**Y Zerbib**)

One nonvoting representative from Hospitals Department in the French Health Ministry.

## STANDARDS, OPTIONS AND RECOMMANDATIONS: LIST OF PUBLICATIONS

Available at: http://www.fnclcc.fr/

## METHODOLOGY

Fervers B, Hardy J, Blanc-Vincent MP, Theobald S, Bataillard A, Farsi F, Gory G, Debuiche S, Guillo S, Renaud-Salis JL, Pinkerton R, Bey P, Philip T (2001) SOR: project methodology. *Br J Cancer* **84** (Suppl 2): 8–16

Fervers B, Hardy J, Blanc-Vincent MP, Theobald S, Bataillard A, Farsi F, Gory-Delabaere G, Debuiche S, Guillo S, Renaud-Salis JL, Pinkerton R, Bey P, Philip T (2001) SOR: project methodology [online]. *Electronic J Oncol* **1**: 1–12. Available: URL: http://www.elecjoncol.org

Bey P, Blanc-Vincent MP, Bonichon F, Farsi F, Fervers B, Labrèze L, Lehmann M, Philip T, Renaud-Salis JL, Sarradet A, Theobald S (1998) Méthodologie d'élaboration des standards, options et recommandations diagnostiques et thérapeutiques en cancérologie de la Fédération Nationale des Centres de Lutte Contre le Cancer (ed). In *Recommandations pour la pratique clinique en cancérologie [CD-ROM]* Fédération Nationale des Centres de Lutte Contre le Cancer (ed). 2nd edn. Paris: FNCLCC, John Libbey EUROTEXT. Standards, Options et Recommandations.

Fervers B, Bonichon F, Demard F, Heron JF, Mathoulin S, Philip T, Renaud-Salis JL, Toma C, Bey P (1995) Méthodologie de développement des standards, options et recommandations diagnostiques et thérapeutiques en cancérologie. *Bull Cancer* **82**: 761–767.

## CANCER IN ADULTS

### Gynaecological malignancies

#### Cancer of the endometrium

Brémond A, Bataillard A, Thomas L, Achard JL, Fervers B, Fondrinier E, Lansac J, Bailly C, Hoffstetter S, Basuyau JP, D'Anjou J, Descamps P, Farsi F, Guastalla JP, Laffargue F, Rodier JF, Vincent P, Pigneux J (2002) Standards, Options et Recommandations 2000: cancer de l'endomètre, stades non métastatiques (rapport abrégé). *Bull Cancer* **89**: 697–706

Brémond A, Bataillard A, Thomas L, Achard JL, Fervers B, Fondrinier E, Lansac J, Bailly C, Hoffstetter S, Basuyau JP, D'Anjou J, Descamps P, Farsi F, Guastalla JP, Laffargue F, Rodier JF, Vincent P, Pigneux J (2002) Standards, Options et Recommandations 2000: cancer de l'endomètre, stades non métastatiques (rapport abrégé). *Gynecol, Obstet et Fertilite* **30**: 902–916

Brémond A, Bataillard A, Thomas L, Achard JL, Fervers B, Fondrinier E, Lansac J, Bailly C, Hoffstetter S, Basuyau JP, D'Anjou J, Descamps P, Farsi F, Guastalla JP, Laffargue F, Rodier JF, Vincent P, Pigneux J (2001) Cancer of the endometrium. *Br J Cancer* **84**(Suppl 2): 31–36

Brémond A, Bataillard A, Thomas L, Achard JL, Fervers B, Fondrinier E, Lansac J, Bailly C, Hoffstetter S, Basuyau JP, D'Anjou J, Descamps P, Farsi F, Guastalla JP, Laffargue F, Rodier JF, Vincent P, Pigneux J (2001) SOR Cancer of the endometrium [online]. *Electron J Oncol* **1**: 23–31. Available: URL: http://www.elecjoncol.org/

Brémond A, Bataillard A, Thomas L, Achard JL, Fervers B, Fondrinier E, Lansac J, Bailly C, Hoffstetter S, Basuyau JP, D'Anjou J, Descamps P, Farsi F, Guastalla JP, Laffargue F, Rodier JF, Vincent P, Pigneux J (2001) Standards, Options et recommandations pour la prise en charge chirurgicale des patientes atteintes de cancer de l'endomètre. *Bull Cancer* **88**(2): 181–198

Brémond A, Bataillard A, Thomas L, Achard JL, Fervers B, Fondrinier E, Lansac J, Bailly C, Hoffstetter S, Basuyau JP, D'Anjou J, Descamps P, Farsi F, Guastalla JP, Laffargue F, Rodier JF, Vincent P, Pigneux J (2001) *Cancer de l'endomètre. Stades non métastatiques*, Vol. 11. Paris: John Libbey EUROTEXT. Standards, Options et Recommandations

Brémond A, Bataillard A, Thomas L, Achard JL, Fervers B, Fondrinier E, Lansac J, Bailly C, Hoffstetter S, Basuyau JP, D'Anjou J, Descamps P, Farsi F, Guastalla JP, Laffargue F, Rodier JF, Vincent P, Pigneux J (2001) In *Standards, Options and Recommandations: Cancer of the Endometrium [Online]*, Fédération Nationale des Centres de Lutte Contre le Cancer (ed) décembre 2001. Available: URL: http://www.fnclcc.fr/-sci/sor/eng_pdf/sor_cancer_endometrium.pdf

Brémond A, Bataillard A, Thomas L, Achard JL, Fervers B, Fondrinier E, Lansac J, Bailly C, Hoffstetter S, Basuyau JP, D'Anjou J, Descamps P, Farsi F, Guastalla JP, Laffargue F, Rodier JF, Vincent P, Pigneux J (2001) In *Standards, Options et Recommandations: Cancer de l'endomètre. Stades non métastatiques [online]*, Fédération Nationale des Centres de Lutte Contre le Cancer (ed) novembre 2001. Available: URL: http://www.fnclcc.fr/-sci/sor/pdf/endometre_integrale_0100.pdf

Brémond A, Bataillard A, Thomas L, Achard JL, Fervers B, Fondrinier E, Lansac J, Bailly C, Hoffstetter S, Basuyau JP, D'Anjou J, Descamps P, Farsi F, Guastalla JP, Laffargue F, Rodier JF, Vincent P, Pigneux J (2001) In *Standards, Options et Recommandations: cancer de l'endométre. Stades non métastatiques [online]*, Fédération Nationale des Centres de Lutte Contre le Cancer (ed) octobre 2001. Available: URL: http://www.fnclcc.fr/-sci/sor/pdf/endometre_abregee_0100.pdf

Thomas L, Bataillard A, Bremond A, Fondrinier E, Fervers B, Achard JL, Lansac J, Bailly C, Hoffstetter S, Basuyau JP, D'Anjou J, Descamps P, Farsi F, Guastalla JP, Laffargue F, Rodier JF, Vincent P, Pigneux J (2001) Standards, Options et Recommandations pour la radiothérapie des patientes atteintes de cancer de l'endomètre. *Cancer Radiother* **5**: 163–192

#### Ovarian cancer

Kerbrat P, Lhommé C, Fervers B, Guastalla JP, Thomas L, Tournemaine N, Basuyau JP, Cohen-Solal C, Duvillard P, Bachelot T, Ray I, Voog E, Dauplat J (2001) In *Standards, Options, Recommandations: Ovarian Cancer [online]*, Fédération Nationale des Centres de Lutte Contre le Cancer (ed) décembre 2001. Available: URL: http://www.fnclcc.fr/-sci/sor/eng_pdf/sor_cancer_ovarian.pdf

Kerbrat P, Lhomme C, Fervers B, Guastalla JP, Thomas L, Tournemaine N, Basuyau JP, Cohen-Solal C, Duvillard P, Bachelot T, Ray I, Voog E, Dauplat J (2001) SOR ovarian cancer [online]. *Electron J Oncol* **1**: 32–42. Available: URL: http://www.elecjoncol.org/

Kerbrat P, Lhommé C, Fervers B, Guastalla JP, Thomas L, Basuyau JP, Duvillard P, Cohen-Solal C, Dauplat J, Tournemaine N, Bachelot T, Ray I, Voog E (2001) Standards, options et recommandations pour la prise en charge initiate des patientes atteintes de tumeurs épithéliales malignes de l'ovaire. *Gynecol Obstet Fertil* **29**: 733–742

Kerbrat P, Lhommé C, Fervers B, Guastalla JP, Thomas L, Basuyau JP, Duvillard P, Cohen-Solal C, Dauplat J, Tournemaine N, Bachelot T, Ray I, Voog E (2001) Standards, options et recommandations pour la prise en charge initiate des patientes atteintes de tumeurs épithéliales malignes de l'ovaire. Seconde partie. *Gynecol Obstet Fertil* **29**: 853–866

Kerbrat P, Lhomme C, Fervers B, Guastalla JP, Thomas L, Tournemaine N, Basuyau JP, Cohen-Solal C, Duvillard P, Bachelot T, Ray I, Voog E, Dauplat J (2001) Ovarian cancer. *Br J Cancer* **84** (Suppl 2): 18–23

Kerbrat P, Lhommé C, Fervers B, Guastalla JP, Thomas L, Basuyau JP, Duvillard P, Cohen-Solal C, Dauplat J, Tournemaine N (2001) Standards, Options et Recommandations pour la prise en charge initiate des patientes atteintes de tumeurs épithéliales malignes de l'ovaire. Feuill Radiol **41**: 272–283

Kerbrat P, Lhommé C, Fervers B, Guastalla JP, Thomas L, Basuyau JP, Duvillard P, Cohen-Solal C, Dauplat J, Tournemaine N (2000) Standards, options et Recommandations pour la prise en charge initiate des patientes atteintes de tumeurs épithéliales malignes de l'ovaire. *Presse Med* **29**: 2116–2127

Kerbrat P, Fervers B, Basuyau JP, Cohen-Solal C, Dauplat J, Duvillard P, Guastalla JP, Lhommé C, Thomas L, Tournemaine N, Société Française d'Oncologie Gynécologique (2000) In *Standards, Options et Recommandations pour la prise en charge initiale des patientes atteintes de tumeurs épithéliales malignes de l'ovaire [online]*. Fédération Nationale des Centres de Lutte Contre le Cancer (ed) novembre 2000. Available: URL: http://www.fnclcc.fr/-sci/sor/pdf/ovaire_abregee_0699.pdf

Kerbrat P, Fervers B, Basuyau JP, Cohen-Solal C, Dauplat J, Duvillard P, Guastalla JP, Lhommé C, Thomas L, Tournemaine N (1998) Fédération Nationale des Centres de Lutte Centre le Cancer, Société Française d'Oncologie Gynécologique. *Tumeurs épithéliales malignes de* l'ovaire, Vol. 4. Paris: John Libbey EUROTEXT. Standards, Options et Recommandations

Kerbrat P, Fervers B, Basuyau JP, Cohen-Solal C, Dauplat J, Duvillard P, Guastalla JP, Lhommé C, Thomas L, Tournemaine N (1998) Standards, options et recommandations pour la prise en charge initiale des patientes atteintes de tumeurs épithéliales malignes de l'ovaire. In *Recommandations pour la pratique clinique en cancérologie [CD-ROM]*, Fédération Nationale des Centres de Lutte Contre le Cancer (ed) 2nd ed. Paris: FNCLCC, John Libbey EUROTEXT; 1998. Standards, Options et Recommandations

#### Cancer of the cervix

Resbeut M, Fondrinier E, Fervers B, Haie-Meder C, Bataillard A, Lhomme C, Asselain B, Basuyau JP, Bremond A, Castaigne D, Dubois JB, Houvenaeghel G, Lartigau E, Leblanc E, Sastre-Garau X, Ternier F, Sarradet A, Guastalla JP, Chauvergne J (2002) Standards, options et recommandations pour la prise en charge de patientes atteintes de cancer invasif du col utérin (Stade non métastatique), version abrégée. *Gynecol Obstet Fertil* **30**: 631–648

Resbeut M, Fondrinier E, Fervers B, Haie-Meder C, Bataillard A, Lhommé C, Asselain B, Basuyau JP, Brémond A, Castaigne D, Dubois JB, Houvenaeghel G, Lartigau E, Leblanc E, Sastre-Garau X, Ternier F, Guastalla JP, Chauvergne J (2001). In *Standards, Options and Recommandations: Carcinoma of the Cervix [Online]*, Fédération Nationale des Centres de Lutte Contre le Cancer (ed) décembre 2001. Available: URL: http://www.fnclcc.fr/-sci/sor/eng_pdf/sor_cancer_cervix.pdf

Resbeut M, Fondrinier E, Fervers B, Haie-Meder C, Bataillard A, Lhomme C, Asselain B, Basuyau JP, Bremond A, Castaigne D, Dubois JB, Houvenaeghel G, Lartigau E, Leblanc E, Sastre-Garaud X, Ternier F, Guastalla JP, Chauvergne J (2001) Carcinoma of the cervix. *Br J Cancer* **84** (Suppl 2): 24–30

Resbeut M, Fondrinier E, Fervers B, Haie-Meder C, Bataillard A, Lhomme C, Asselain B, Basuyau JP, Bremond A, Castaigne D, Dubois JB, Houvenaeghel G, Lartigau E, Leblanc E, Sastre-Garaud X, Ternier F, Guastalla JP, Chauvergne J (2001) SOR: carcinoma of the cervix [online]. *Electron J Oncol* **1**: 13–22. Available: URL: http://www.elecjoncol.org/

Haie-Meder C, Fervers B, Chauvergne J, Fondrinier E, Lhommé C, Bataillard A, Guastalla JP, Resbeut M, Asselain B, Basuyau JP, Brémond A, Castaigne D, Dubois JB, Houvenaeghel G, Lartigau E, Leblanc E, Sastre-Garau X, Ternier F (2000) In *Radiochimiothérapie concommitante dans les cancers du col de l'utérus: analyse critique des données et mise à jour des standards*, options et recommandations [online], Fédération Nationale des Centres de Lutte Contre le Cancer (ed) November 2000. Available: URL: http://www.fnclcc.fr/-sci/sor/pdf/uterus_radiochimiotherapie_integrale_0699.pdf

Resbeut M, Fondrinier E, Fervers B, Asselain B, Basuyau JP, Brémond A, Castaigne D, Chauvergne J, Dubois JB, Haie-Meder C, Houvenaeghel G, Lartigau E, Leblanc E, Sarradet A, Sastre-Garau X, Ternier F, Société Française d'Oncologie Gynécologique (2000) *Standards, Options et Recommandations pour la prise en charge des patientes atteintes de cancers invasifs du col utérin (stades non métastatiques) [online]*, Fédération Nationale des Centres de Lutte Contre le Cancer (ed) novembre 2000. Available: URL: http://www.fnclcc.fr/-sci/sor/pdf/uterus_abregee_0400.pdf

Haie-Meder C, Lhomme C, de Crevoisier R, Morice P, Resbeut M (2000) Radiochimiothérapie concomitante dans les cancers du col de l'utérus: modifications des standards. *Cancer Radiother* **4** (Suppl 1): 134s-140s

Haie-Meder C, Fervers B, Chauvergne J, Fondrinier E, Lhommé C, Guastalla JP, Resbeut M (1999) Radiochimiothérapie concommitante dans les cancers du col de l'utérus: analyse critique des donnees et mise à jour des standards, options et recommandations. *Bull Cancer* **86**: 829–841.

Resbeut M, Fondrinier E, Fervers B, Asselain B, Basuyau JP, Brémond A, Castaigne D, Chauvergne J, Dubois JB, Haie-Meder C, Houvenaeghel G, Lartigau E, Leblanc E, Sarradet A, Sastre-Garau X, Ternier F, Fédération Nationale des Centres de Lutte Contre le Cancer, Société Française d'Oncologie Gynécologique (1999) *Cancers invasifs du col utérin: stades non métastatiques*, Vol. 9. Paris: John Libbey EUROTEXT. Standards, Options et Recommandations

Resbeut M, Fondrinier E, Fervers B, Asselain B, Basuyau JP, Brémond A, Castaigne D, Chauvergne J, Dubois JB, Haie-Meder C, Houvenaeghel G, Lartigau E, Leblanc E, Sarradet A, Sastre-Garau X, Ternier F (1998) Standards, options et recommandations pour la prise en charge des patientes atteintes de cancers invasifs du col utérin (stades non métastatiques). In *Recommandations pour la pratique clinique en cancérologie [CD-ROM]*, Fédération Nationale des Centres de Lutte Contre le Cancer (ed) 2nd edn. Paris: FNCLCC, John Libbey EUROTEXT. Standards, Options et Recommandations

### Nonmetastatic breast cancer

Fourquet A, Cutuli B, Luporsi E, Mauriac L, Garbay JR , Giard S, Spyratos F, Sigal-Zafrani B, Dilhuydy JM, Acharian V, Balu-Maestro C, Blanc-Vincent MP, Cohen-Solal C, De Lafontan B, Dilhuydy MH, Duquesne B, Gilles R, Lesur A, Shen N, Cany L, Dagousset I, Gaspard MH, Hoarau H, Hubert A, Monira MH, Perrié N, Romieu G (2002) Standards, Options et Recommandations 2001 pour la radiothérapie des patientes atteintes d'un cancer du sein infiltrant non métastatique, mise à jour. *Cancer Radiother* **6**: 238–258

Mauriac L, Luporsi E, Cutuli B, Fourquet A, Garbay JR, Giard S, Spyratos F, Sigal-Zafrani B, Dilhuydy JM, Acharian V, Balu-Maestro C, Blanc-Vincent MP, Cohen-Solal C, De Lafontan B, Dilhuydy MH, Duquesne B, Gilles R, Lesur A, Shen N (2002) Standards, Options et Recommandations: cancers du sein infiltrants non métastiques (2e édition, mise à jour, version abrégée). *Bull Cancer* **89**: 207–224

Mauriac L, Luporsi E, Cutuli B, Garbay JR, Giard S, Spyratos F, Sigal-Zafrani B, Dilhuydy JM, Acharian V, Balu-Maestro C, Blanc-Vincent MP, Cohen-Solal C, De Lafontan B, Dilhuydy MH, Duquesne B, Gilles R, Lesur A, Shen N, Cany L, Dagousset I, Gaspard MH, Hoarau H, Hubert A, Monira MH, Perrié N, Romieu G (2001) In Cancers du sein infiltrants non métastatiques, Fédération Nationale des Centres de Lutte Contre le Cancer (ed) Vol. 12. Paris: John Libbey EUROTEXT. Standards, Options et Recommandations

Mauriac L, Luporsi E, Cutuli B, Fourquet A, Garbay JR, Giard S, Spyratos F, Sigal-Zafrani B, Dilhuydy JM, Acharian V, Balu-Maestro C, Blanc-Vincent MP, Cohen-Solal C, De Lafontan B, Dilhuydy MH, Duquesne B, Gilles R, Lesur A, Shen N (2001) In *Standards, Options et Recommandations: cancers du sein infiltrants non métastatiques [online]*, Fédération Nationale des Centres de Lutte Contre le Cancer (ed) décembre 2001. Available: URL: http://www.fnclcc.fr/-sci/sor/pdf/sein_abregee_0101.pdf

Mauriac L, Luporsi E, Cutuli B, Fourquet A, Garbay JR, Giard S, Spyratos F, Sigal-Zafrani B, Dilhuydy JM, Acharian V, Balu-Maestro C, Blanc-Vincent MP, Cohen-Solal C, De Lafontan B, Dilhuydy MH, Duquesne B, Gilles R, Lesur A, Shen N (2001) *Standards, Options et Recommandations: cancers du sein infiltrants non métastatiques. [online]*, Fédération Nationale des Centres de Lutte Contre le Cancer (ed) November 2001. Available: URL: http://www.fnclcc.fr/-sci/sor/pdf/sein_integrale_0101.pdf

Mauriac L, Luporsi E, Blanc-Vincent MP, Dilhuydy JM, Acharian V, Balu-Maestro C, Cohen-Solal C, Cutuli B, Dilhuydy MH, Duquesne B, Fourquet A, Garbay JR, Giard S, Gilles R, Lesur A, Shen N, Spyratos F, Sigal-Zafrani B (2001) In *Standards, Options et Recommandations: cancers du sein non métastatiques. Arbres de décision [online]*, Fédération Nationale des Centres de Lutte Contre le Cancer (ed) décembre 2001. Available: URL: http://www.fnclcc.fr/-sci/sor/pdf/sein_arbres_0101.pdf

Mauriac L, Blanc-Vincent MP, Luporsi E, Cutuli B, Fourquet A, Garbay JR, Giard S, Spyratos F, Zafrani B, Dilhuydy JM (2000) Standards, Options et Recommandations (SOR): hormonothérapie dans les cancers du sein non métastatiques. *Bull Cancer* **87**: 469–490

Mauriac L, Asselain B, Blanc-Vincent MP, Cutuli B, Dilhuydy JM, Fourquet A, Garbay JR, Luporsi E, Spyratos F, Zafrani B (1996) In *Cancers du sein non métastatiques*, Fédération Nationale des Centres de Lutte Contre le Cancer (ed) Vol. 3. Paris: Arnette Blackwell; 1996. Standards, Options et Recommandations

Blanc-Vincent MP, Mauriac L, Asselain B, Cutuli B, Dilhuydy JM, Fourquet A, Garbay JR, Luporsi E, Spyratos F, Zafrani B (1999) Recommandations pour la pratique clinique en cancérologie: ‘Standards, Options et Recommandations’: Cancer du sein non métastatique. La lettre de l'ANHP March 1999

Mauriac L, Asselain B, Blanc-Vincent MP, Cutuli B, Dilhuydy JM, Fourquet A, Garbay JR, Luporsi E, Spyratos F, Zafrani B *Recommandations pour la pratique clinique en cancérologie [CD-ROM]*, (1998) Standards, options et recommandations pour les cancers du sein non métastatiques. In Fédération Nationale des Centres de Lutte Contre le Cancer (ed) 2nd edn. Paris: FNCLCC, John Libbey EUROTEXT. Standards, Options et Recommandations

Mauriac L, Asselain B, Blanc-Vincent MP, Cutuli B, Dilhuydy JM, Fourquet A, Garbay JR, Luporsi E, Spyratos F, Zafrani B (1997). In *Standards, Options et Recommandations pour le cancer du sein non métastatique [CD-ROM]*, Fédération Nationale des Centres de Lutte Contre le Cancer (ed). Paris: FNCLCC

### Gastrointestinal malignancies

#### Colon cancer

Adenis A, Conroy T, Lasser P, Merrouche Y, Monges G, Rivoire M, Rougier P, Ruggieri-Pignon S (2001) SOR carcinoma of the colon [online]. *Electron J Oncol* **1**: 83–89. Available: URL: http://www.elecjoncol.org/

Adenis A, Conroy T, Lasser P, Merrouche Y, Monges G, Rivoire M, Rougier P, Ruggieri-Pignon S (2001) Carcinoma of the colon. *Br J Cancer* **84** (Suppl 2): 65–68

Adenis A, Conroy T, Lasser P, Merrouche Y, Monges G, Rivoire M, Rougier P, Ruggieri-Pignon S. In *Standards, Options and Recommandations: carcinoma of the colon [online]*, Fédération Nationale des Centres de Lutte Contre le Cancer (ed) November 2001. Available: URL: http://www.fnclcc.fr/-sci/sor/eng_pdf/sor_cancer_colon.pdf

Adenis A, Conroy T, Lasser P, Merrouche Y, Monges G, Rivoire M, Rougier P, Ruggieri-Pignon S (2000) In *Standards, Options et Recommandations pour la prise en charge des patients attaints de cancer du côlon [online]*, Fédération Nationale des Centres de Lutte Contre le Cancer (ed) November 2000. Available: URL: http://www.fnclcc.fr/-sci/sor/pdf/colon_abregee_0300.pdf

Adenis A, Conroy T, Lasser P, Merrouche Y, Monges G, Rivoire M, Rougier P, Ruggieri-Pignon S, Sarradet A, Toma C (1998) In Standards, options et recommandations pour la prise en charge des patients atteints de cancer du côlon. In *Recommandations pour la pratique clinique en cancérologie [CD-ROM]*, Fédération Nationale des Centres de Lutte Contre le Cancer (ed) 2nd edn. Paris: FNCLCC, John Libbey EUROTEXT. Standards, Options et Recommandations

Conroy T, Adenis A (1998) Standards, options et recommandations pour la surveillance après traitement d'un cancer du côlon. *Bull Cancer* **85**: 152–159

Adenis A, Conroy T, Lasser P, Merrouche Y, Monges G, Rivoire M, Rougier P, Ruggieri-Pignon S, Toma C (1995) Standards, options et recommandations pour la prise en charge des patients atteints de cancer du côlon. In Cancers digestifs: cancer de l'oesophage, carcinome hépato- cellulaire, cancer du côlon, Fédération Nationale des Centres de Lutte Contre le Cancer, (ed) Vol. 2, pp 85–160. Paris: Arnette Blackwell. Standards, Options et Recommandations

#### Hepatocellular carcinoma

Giovannini M, Elias D, Monges G, Raoul JL, Rougier P (2001) Hepatocellular carcinoma. *Br J Cancer* **84** (Suppl 2): 74–77

Giovannini M, Elias D, Monges G, Raoul JL, Rougier P (2001) SOR hepatocellular carcinoma [online]. *Electron J Oncol* **1**: 64–69. Available: URL: http://www.elecjoncol.org/

Giovannini M, Elias D, Monges G, Raoul JL, Rougier P, Sarradet A (2001) In *Standards, Options and Recommandations: hepatocellular carcinoma [online]*, Fédération Nationale des Centres de Lutte Contre le Cancer (ed) December 2001. Available: URL: http://www.fnclcc.fr/-sci/sor/eng_pdf/sor_hepatocellular_carcinoma.pdf

Elias D, Giovannini M, Monges G, Raoul JL, Rougier P, Sarradet A (2000) In *Standards, Options et Recommandations pour la prise en charge des patients atteints de carcinomes hépato-cellulaires [online]*, Fédération Nationale des Centres de Lutte Contre le Cancer (ed) November 2000. Available: URL: http://www.fnclcc.fr/-sci/sor/pdf/chc_abregee_0300.pdf

Giovannini M, Elias D, Monges G, Raoul JL, Rougier P, Sarradet A, Toma C (1998) Standards, options et recommandations pour la prise en charge des patients atteints de carcinomes hépato-cellulaires. In *Recommandations pour la pratique clinique en cancérologie [CD-ROM]*, Fédération Nationale des Centres de Lutte Contre le Cancer (ed) 2nd edn. Paris: FNCLCC, John Libbey EUROTEXT; 1998. Standards, Options et Recommandations

Giovannini M, Elias D, Monges G, Raoul JL, Rougier P, Toma C (1995) Standards, options et recommandations pour la prise en charge des patients atteints de carcinomes hépato-cellulaires. In *Cancers digestifs: cancer de l'oesophage, carcinome hépato- cellulaire, cancer du côlon*, Fédération Nationale des Centres de Lutte Contre le Cancer (ed) Vol. 2, pp 39–83. Paris: Arnette Blackwell. Standards, Options et Recommandations

#### Oesophageal cancer

Seitz JF, Sarradet A, Francois E, Jacob JH, Ollivier JM, Rougier P, Roussel A (2001). SOR Carcinoma of the oesophagus [online]. *Electron J Oncol* **1**: 78–82. Available: URL: http://www.elecjoncol.org/

Seitz JF, Sarradet A, Francois E, Jacob JH, Ollivier JM, Rougier P, Roussel A (2001) Carcinoma of the oesophagus. *Br J Cancer* **84** (Suppl 2): 61–64

Seitz JF, Sarradet A, Francois E, Jacob JH, Ollivier JM, Rougier P, Roussel A, Conroy T (2001) In *Standards, Options, Recommandations: carcinoma of the oesophagus [online]*, Fédération Nationale des Centres de Lutte Contre le Cancer (ed) December 2001. Available: URL: http://www.fnclcc.fr/-sci/sor/eng_pdf/sor_cancer_oesophagus.pdf

Seitz JF, Sarradet A, Francois E, Jacob JH, Ollivier JM, Rougier P, Roussel A, Conroy T (2000) In *Standards, Options et Recommandations pour la prise en charge des patients atteints de cancer de l'oesophage [online]*, Fédération Nationale des Centres de Lutte Contre le Cancer (ed) November 2000. Available: URL: http://www.fnclcc.fr/-sci/sor/pdf/oesophage_abregee_0300.pdf

Seitz JF, François E, Jacob JH, Ollivier JM, Rougier P, Roussel A, Sarradet A, Toma C (1998) Standards, options et recommandations pour la prise en charge des patients atteints de cancer de l'oesophage. In *Recommandations pour la pratique clinique en cancérologie [CD-ROM]*, Fédération Nationale des Centres de Lutte Contre le Cancer (ed) 2nd edn. Paris: FNCLCC, John Libbey EUROTEXT. Standards, Options et Recommandations

Seitz JF, François E, Ollivier JM, Rougier P, Roussel A, Toma C (1995) Standards, options et recommandations pour la prise en charge des patients atteints de cancer de l'oesophage. In *Cancers digestifs: cancer de l'oesophage, carcinome hépato-cellulaire, cancer du côlon*, Fédération Nationale des Centres de Lutte Contre le Cancer (ed) Vol. 2, pp 1–37. Paris: Arnette Blackwell. Standards, Options et Recommandations

#### Cancer of the pancreas

Ducreux M, François E, Saint-Aubert B, NGuyen TD, Sarradet A, Rougier P (1998) Cancer du pancreas. In *Cancers digestifs II: pancréas et rectum*, Fédération Nationale des Centres de Lutte Contre le Cancer (ed) Vol. 5, pp 3–77. Paris: John Libbey EUROTEXT. Standards, Options et Recommandations

Ducreux M, François E, Saint-Aubert B, NGuyen TD, Sarradet A, Rougier P (1998) Standards, options et recommandations pour la prise en charge des patients atteints de cancer du pancreas. In *Recommandations pour la pratique clinique en cancérologie [CD-ROM]*, Fédération Nationale des Centres de Lutte Contre le Cancer (ed) 2nd edn. Paris: FNCLCC, John Libbey EUROTEXT. Standards, Options et Recommandations

#### Cancer of the rectum

Bécouarn Y, Blanc-Vincent MP, Ducreux M, Lasser P, Dubois JB, Giovannini M, Rougier P (2001) Cancer of the rectum. *Br J Cancer* **84** (Suppl 2): 69–73

Bécouarn Y, Blanc-Vincent MP, Ducreux M, Lasser P, Dubois JB, Giovannini M, Rougier P (2001) SOR Cancer of the rectum [online]. *Electron J Oncol* **1**: 70–77. Available: URL: http://www.elecjoncol.org/

Bécouarn Y, Blanc-Vincent MP, Lasser P, Dubois JB, Ducreux M, Giovannini M, Rougier P (2000) In *Standards, Options et Recommandations pour la prise en charge des patients atteinds d'adénocarcinome primitif du rectum [online]*, Fédération Nationale des Centres de Lutte Contre le Cancer (ed) November 2000. Available: URL: http://www.fnclcc.fr/-sci/sor/pdf/rectum_abregee_1298.pdf

Bécouarn Y, Blanc-Vincent MP, Lasser P, Dubois JB, Ducreux M, Giovannini M, Rougier P (1999) Standards, Options et Recommandations pour la prise en charge des patients atteints d'adénocarcinome primitif du rectum. *Presse Med* **28**: 1367–1377

Bécouarn Y, Blanc-Vincent MP, Lasser P, Dubois JB, Ducreux M, Giovannini M, Rougier P (1998) Adénocarcinome primitif du rectum. In *Cancers digestifs II: pancréas et rectum*, Fédération Nationale des Centres de Lutte Contre le Cancer (ed) Vol. 5, pp 79–229. Paris: John Libbey EUROTEXT. Standards, Options et Recommandations

Bécouarn Y, Blanc-Vincent MP, Lasser P, Dubois JB, Ducreux M, Giovannini M, Rougier P (1998) Standards, options et recommandations pour la prise en charge des patients atteints d'adénocarcinome primitif du rectum. In *Recommandations pour la pratique clinique en cancérologie [CD-ROM]*, Fédération Nationale des Centres de Lutte Contre le Cancer (ed) 2nd edn. Paris: FNCLCC, John Libbey EUROTEXT. Standards, Options et Recommandations

### Haematological malignancies

#### Cerebral lymphoma

Blay JY, Farsi F, Carrie C, Biron P, Fervers B, Philip T (1999) Lymphomes malins non hodgkiniens cérébraux primitifs en dehors de l'infection par VIH. In *Maladie de Hodgkin chez l'adulte. Lymphomes malins non hodgkiniens cérébraux en dehors de l'infection par VIH*, Fédération Nationale des Centres de Lutte Contre le Cancer (ed) Vol. 7, pp 169–217. Paris: John Libbey EUROTEXT Standards, Options et Recommandations

Blay JY, Farsi F, Carrie C, Biron P, Fervers B, Philip T (1998) Standards, options et recommandations pour la prise en charge des patients atteints de lymphomes malins non hodgkiniens cérébraux primitifs en dehors de l'infection par VIH. In *Recommandations pour la pratique clinique en cancérologie [CD-ROM]*, Fédération Nationale des Centres de Lutte Contre le Cancer (ed) 2nd edn. Paris: FNCLCC, John Libbey EUROTEXT. Standards, Options et Recommandations

#### Hodgkin's disease

Fermé C, Cosset JM, Fervers B, Sebban C, Cutuli B, Henry-Amar M, Stines J, Giammarile F, Bey P, Carella AM, Philip T (2001) Hodgkins disease. *Br J Cancer* **84** (Suppl 2): 55–60

Fermé C, Cosset JM, Fervers B, Sebban C, Cutuli B, Henry-Amar M, Stines J, Giammarile F, Bey P, Carella AM, Philip T (2001) SOR Hodgkins disease [online]. *Electron J Oncol* **1**: 90–98. Available: URL: http://www.elecjoncol.org/

Fermé C, Cosset JM, Fervers B, Sebban C, Cutuli B, Henry-Amar M, Stines J, Giammarile F, Bey P, Carella AM, Philip T (2001) In *Standards, Options and Recommandations: Hodgkins Disease [Online]*, Fédération Nationale des Centres de Lutte Contre le Cancer (ed) December 2001. Available: URL: http://www.fnclcc.fr/-sci/sor/eng_pdf/sor_cancer_hodgkins.pdf

Fermé C, Cosset JM, Fervers B, Sebban C, Cutuli B, Henry-Amar M, Stines J, Giammarile F, Bey P, Carella AM, Philip T (2000) In *Standards, Options et Recommandations pour la prise en charge des patients adultes atteints de la maladie de Hodgkin [online]*, Fédération Nationale des Centres de Lutte Contre le Cancer (ed) November 2000. Available: URL: http://www.fnclcc.fr/-sci/sor/pdf/hodgkin_abregee_0300.pdf

Fermé C, Cosset JM, Sebban C, Fervers B, Cutuli B, Henry-Amar M, Stines J, Giammarile F, Bey P, Carella AM, Philip T, Groupe d'Etude des Lymphomes de l'Adulte. Maladie de Hodgkin chez l'adulte (1999). In *Maladie de Hodgkin chez l'adulte. Lymphomes malins non hodgkiniens cérébraux primitifs en dehors de l'infection par VIH*, Fédération Nationale des Centres de Lutte Contre le Cancer (ed) Vol. 7, pp 1–166. Paris: John Libbey EUROTEXT. Standards, Options et Recommandations

Fermé C, Cosset JM, Fervers B, Sebban C, Cutuli B, Henry-Amar M, Stines J, Giammarile F, Bey P, Carella AM, Philip T (1999) Standards, options et recommandations pour la prise en charge des patients adultes attaints de la maladie de Hodgkin. *Presse Med* **28**: 2233–2245

Fermé C, Cosset JM, Sebban C, Fervers B, Cutuli B, Henry-Amar M, Stines J, Giammarile F, Bey P, Carella AM, Philip T (1998) Standards, options et recommandations pour la prise en charge des patients adultes atteints de la maladie de Hodgkin. In *Recommandations pour la pratique clinique en cancérologie [CD-ROM]*, Fédération Nationale des Centres de Lutte Contre le Cancer (ed) 2nd edn. Paris: FNCLCC, John Libbey EUROTEXT. Standards, Options et Recommandations

### Tumours of the skin and soft tissue

#### Malignant melanoma

Négrier S, Fervers B, Bailly C, Beckendorf V, Cupissol D, Dore JF, Dorval T, Garbay JR, Vilmer C (2001) Cutaneous melanoma. *Br J Cancer* **84** (Suppl 2): 81–85

Négrier S, Fervers B, Bailly C, Beckendorf V, Cupissol D, Dore JF, Dorval T, Garbay JR, Vilmer C (2)001 SOR Cutaneous melanoma [online]. *Electron J Oncol* **1**: 99–106. Available: URL: http://www.elecjoncol.org/

Négrier S, Fervers B, Bailly C, Beckendorf V, Cupissol D, Doré JF, Dorval T, Garbay JR, Vilmer C (2001) In *Standards, Options and Recommandations: Cutaneous Melanoma [online]*, Fédération Nationale des Centres de Lutte Contre le Cancer (ed) December 2001. Available: URL: http://www.fnclcc.fr/-sci/sor/eng_pdf/sor_melanoma.pdf

Négrier S, Fervers B, Bailly C, Beckendorf V, Cupissol D, Doré JF, Dorval T, Garbay JR, Vilmer C (2001) In Standards, Options et Recommandations pour la prise en charge des patients atteints de mélanome cutané [online], Fédération Nationale des Centres de Lutte Contre le Cancer (ed) September 2001. Available: URL: http://www.fnclcc.fr/-sci/sor/pdf/melanome_integrale_1299.pdf

Négrier S, Fervers B, Bailly C, Beckendorf V, Cupissol D, Doré JF, Dorval T, Garbay JR, Vilmer C (2000) Standards, options et recommandations pour la prise en charge des patients atteints de mélanome cutané. *Bull Cancer* **87**: 173–182

Négrier S, Fervers B, Bailly C, Beckendorf V, Cupissol D, Doré JF, Dorval T, Garbay JR, Vilmer C (2000) In *Standards, Options et Recommandations pour la prise en charge des patients atteints de mélanome cutané [online]*, Fédération Nationale des Centres de Lutte Contre le Cancer (ed) November 2000. Available: URL: http://www.fnclcc.fr/-sci/sor/pdf/melanome_abregee_1299.pdf

Négrier S, Fervers B, Bailly C, Beckendorf V, Cupissol D, Doré JF, Dorval T, Garbay JR, Vilmer C (2000) Standards, Options et Recommandations pour la prise en charge des patients atteints de mélanome cutané. *Presse Med* **29**: 1317–1326

Négrier S, Fervers B, Bailly C, Beckendorf V, Cupissol D, Doré JF, Dorval T, Garbay JR, Vilmer C (1998) In *Mélanome cutané*, Fédération Nationale des Centres de Lutte Contre le Cancer (ed) Vol. 6. Paris: John Libbey EUROTEXT. Standards, Options et Recommandations

Négrier S, Fervers B, Bailly C, Beckendorf V, Cupissol D, Doré JF, Dorval T, Garbay JR, Vilmer C (1998) Standards, options et recommandations pour la prise en charge des patients atteints de mélanome cutané. In: Fédération Nationale des Centres de Lutte Contre le Cancer (ed) *Recommandations pour la pratique clinique en cancérologie [CD-ROM]*, 2nd edn. Paris: FNCLCC, John Libbey EUROTEXT. Standards, Options et Recommandations

#### Soft-tissue sarcoma

Blay JY, Bonichon F, Bui BN, Busson A, Carrie C, Chauvel P, Coindre JM, Cuisenier J, Delannes M, Kantor G, Kind M, Lagarde P, Le Cesne A, Mathoulin S, Ollivier JM, Peiffert D, Rivoire M, Stöckle E, Terrier F (2001) In *Standards, Options et Recommandations pour la prise en charge des patients adultes atteints de sarcomes des tissus mous [online]*, Fédération Nationale des Centres de Lutte Contre le Cancer (ed) September 2001. Available: URL: http://www.fnclcc.fr/-sci/sor/pdf/sarcome_integrale_0595.pdf.

Bui BN, Blay JY, Bonichon F, Busson A, Carrie C, Chauvel P, Coindre JM, Cuisenier J, Delannes M, Kantor G, Kind M, Lagarde P, Le Cesne A, Ollivier L, Peiffert D, Rivoire M, Stöckle E, Terrier P (1998) Standards, options et recommandations pour la prise en charge des patients adultes atteints de sarcomes des tissus mous. In *Recommandations pour la pratique clinique en cancérologie [CD-ROM]*, Fédération Nationale des Centres de Lutte Contre le Cancer (ed) 2nd edn. Paris: FNCLCC, John Libbey EUROTEXT. Standards, Options et Recommandations

Bui BN, Blay JY, Bonichon F, Busson A, Carrie C, Chauvel P, Coindre JM, Cuisenier J, Delannes M, Kantor G, Kind M, Lagarde P, Le Cesne A, Ollivier L, Peiffert D, Rivoire M, Stöckle E, Terrier P (1995) Standards, options et recommandations pour la prise en charge des patients adultes attaints de sarcomes des tissus mous. In *Sarcomes des tissus mous et ostéosarcomes*, Fédération Nationale des Centres de Lutte Contre le Cancer (ed) Vol. 1, pp 1-113. Paris: Arnette Blackwell. Standards, Options et Recommandations

### Cancers of the head and neck

#### Cancers of the oral cavity

Brugére J, Brunin F, Farsi F, Gourmet R, Jaulerry C, Peiffert D, Rodriguez J (1998) Standards, options et recommandations pour la prise en charge des patients atteints de carcinomes épidermoïdes de la cavité buccale. In *Recommandations pour la pratique clinique en cancérologie [CD-ROM]*, Fédération Nationale des Centres de Lutte Contre le Cancer (ed) 2nd edn. Paris: FNCLCC, John Libbey EUROTEXT. Standards, Options et Recommandations

#### Cancer of the salivary glands

Bensadoun RJ, Blanc-Vincent MP, Chauvel P, Dassonville O, Gory-Delabaere G, Demard F (2001). Malignant tumours of the salivary glands. *Br J Cancer* **84** (Suppl 2): 42–48

Bensadoun RJ, Blanc-Vincent MP, Chauvel P, Dassonville O, Gory-Delabaere G, Demard F. In *Standards, Options and Recommandations: Malignant Tumours of the Salivary Glands [Online]*, Fédération Nationale des Centres de Lutte Contre le Cancer (ed) December 2001. Available: URL: http://www.fnclcc.fr/-sci/sor/eng_pdf/sor_cancer_salivaryglands.pdf

Bensadoun RJ, Blanc-Vincent MP, Chauvel P, Dassonville O, Gory-Delabaere G, Demard F (2001) SOR Malignant tumours of the salivary glands [online]. *Electron J Oncol* **1**: 54–63. Available: URL: http://www.elecjoncol.org/

Bensadoun RJ, Blanc-Vincent MP, Chauvel P, Dassonville O, Demard F, Gory-Delabaere G (2000) In *Standards, Options et Recommandations pour les patients atteints de tumeurs malignes des glandes salivaires (lymphomes inclus) [online]*, Fédération Nationale des Centres de Lutte Contre le Cancer (ed) November 2000. Available: URL: http://www.fnclcc.fr/-sci/sor/pdf/glandes_salivaires_abregee_0899.pdf

Bensadoun RJ, Blanc-Vincent MP, Chauvel P, Dassonville O, Demard F (1998) Standards, options et recommandations pour les patients atteints de tumeurs malignes des glandes salivaires (lymphomes inclus). In *Recommandations pour la pratique clinique en cancérologie [CD-ROM]*, Fédération Nationale des Centres de Lutte Contre le Cancer (ed) 2nd edn. Paris: FNCLCC, John Libbey EUROTEXT. Standards, Options et Recommandations

#### Cancers of the oropharynx

Renaud-Salis JL, Blanc-Vincent MP, Brugere J, Demard F, Faucher A, Gory-Delabaere G, Pinsolle J (2001) Epidermoid cancers of the oropharnyx. *Br J Cancer* **84** (Suppl 2): 37–41

Renaud-Salis JL, Blanc-Vincent MP, Brugere J, Demard F, Faucher A, Gory-Delabaere G, Pinsolle J (2001). SOR Epidermoid cancers of the oropharnyx [online]. *Electron J Oncol* **1**: 23–31 Available: URL: http://www.elecjoncol.org/

Renaud-Salis JL, Blanc-Vincent MP, Brugère J, Demard F, Faucher A, Gory-Delabaere G, Pinsolle J (2000) In *Standards, Options et Recommandations pour la prise en charge des patients atteints de cancers épidermoïdes de l'oropharynx [online]*, Fédération Nationale des Centres de Lutte Contre le Cancer (ed) November 2000. Available: URL: http://www.fnclcc.fr/-sci/sor/pdf/oropharynx_integrale_1299.pdf

Renaud-Salis JL, Blanc-Vincent MP, Brugère J, Demard F, Faucher A, Gory-Delabaere G, Pinsolle J (2000) In *Standards, Options et Recommandations pour la prise en charge des patients atteints de cancers épidermoïdes de l'oropharynx [online]*, Fédération Nationale des Centres de Lutte Contre le Cancer (ed) November 2000. Available: URL: http://www.fnclcc.fr/-sci/sor/pdf/oropharynx_abregee_1299.pdf

Renaud-Salis JL, Blanc-Vincent MP, Brugère J, Demard F, Faucher A, Gory-Delabaere G, Pinsolle J (1999) Standards, options et recommandations pour le diagnostic, le traitement et la surveillance des cancers épidermoïdes de l'oropharynx. *Bull Cancer* **86**: 550–572

Renaud-Salis JL, Blanc-Vincent MP, Brugère J, Demard F, Faucher A (1998) Standards, options et recommandations pour le diagnostic, le traitement et la surveillance des cancers épidermoïdes de l'oropharynx. In Recommandations pour la pratique clinique en cancérologie [CD-ROM], Fédération Nationale des Centres de Lutte Contre le Cancer (ed) 2nd edn. Paris: FNCLCC, John Libbey EUROTEXT. Standards, Options et Recommandations

### Urology–Nephrology

*Prostatic cancer*

Pommier P, Villiers A, Bataillard A, Brune D, Fervers B, Bachaud JM, Berger N, Bertrand AF, Bouvier R, Daver A, Fontaine E, Haillot O, Lagrange JL, Molinié V, Muratet JP, Pabot du Chatelard P, Peneau M, Prapotnitch D, Ravery V, Richaud P, Rossi D, Soulié M (2002) Standards, Options et Recommandations pour la radiothérapie externe des patients atteints de cancer de la prostate: évaluation de l'effet dose. *Cancer Radiother* **6**: 119–126

Villers A, Pabot du Chatelard P, Pommier P, Soulié M, Bataillard A, Fervers B, Bachaud JM, Berger N, Bertrand AF, Bouvier R, Brune D, Daver A, Fontaine E, Haillot O, Lagrange JL, Molinié V, Muratet JP, Peneau M, Prapotnitch D, Ravery V, Richaud P, Rossi D (2002) Standards, options et Recommandations 2001 pour la prise en charge des patients atteints de cancer de la prostate non métastatique: éléments de la décision thérapeutique. *Bull Cancer* **89**: 619–634

Pommier M, Villers A, Bataillard A, Brune D, Fervers B, Bachaud JM, Berger N, Bertrand AF, Bouvier R, Daver A, Fontaine E, Guilloneau B, Haillot O, Lagrange JL, Molinié V, Muratet JP, Pabot du Chatelard P, Peneau M, Prapotnitch D, Ravery V, Richaud P, Rossi D, Soret JY (2001) Standards, options et recommandations pour la curiethérapie des patients atteints de cancer de la prostate: efficacité et toxicité. *Cancer Radiother* **6**: 770–786

Villers A, Pommier M, Bataillard A, Fervers B, Bachaud JM, Berger N, Bertrand AF, Bouvier R, Brune D, Daver A, Fontaine E, Haillot O, Lagrange JL, Molinié V, Muratet JP, Pabot du Chatelard P, Peneau M, Prapotnitch D, Ravery V, Richaud P, Rossi D, Soulié M (2002) In *Cancer de la prostate non métastatique*, Fédération Nationale des Centres de Lutte Contre le Cancer (ed) Vol. 14. Paris: John Libbey EUROTEXT. Standards, Options et Recommandations

#### Renal-cell carcinoma

Beckendorf V, Bladou F, Farsi F, Kaemmerlen P, Négrier S, Philip T, Terrier-Lacombe MJ (2000) Standards, options et recommandations pour la radiothérapie du cancer du rein. *Cancer Radiother* **4**(3): 223–233

Beckendorf V, Bladou F, Farsi F, Kaemmerlen P, Négrier S, Philip T, Terrier-Lacombe MJ (2000) In *Standards, Options et Recommandations pour la radiothérapie du cancer du rein [online]*, Fédération Nationale des Centres de Lutte Contre le Cancer (ed) November 2000. Available: URL: http://www.fnclcc.fr/-sci/sor/pdf/rein_radiotherapie_integrale_0400.pdf

Beckendorf V, Bladou F, Farsi F, Kaemmerlen P, Négrier S, Philip T, Terrier-Lacombe MJ (1998) Standards, options et recommandations pour la prise en charge des patients atteints de cancers du rein. In *Recommandations pour la pratique clinique en cancérologie [CD-ROM]*, Fédération Nationale des Centres de Lutte Contre le Cancer (ed) 2nd edn. Paris: FNCLCC, John Libbey EUROTEXT. Standards, Options et Recommandations

### Thymic tumours

Ruffié P, Gory-Delabaere G, Fervers B, Regnard JF, Resbeut M (2001) Epithelial tumours of the thymus. *Br J Cancer* **84** (Suppl 2): 51–54

Ruffié P, Gory-Delabaere G, Fervers B, Regnard JF, Resbeut M (2001) SOR epithelial tumours of the thymus [online]. *Electron J Oncol* **1**: 107–111 Available: URL: http://www.elecjoncol.org/

Ruffié P, Gory-Delabaere G, Fervers B, Regnard JF, Resbeut M (2001) In *Standards, Options and Recommandations: Epithelial Tumours of the Thymus [Online]*, Fédération Nationale des Centres de Lutte Contre le Cancer (ed) December 2001. Available: URL: http://www.fnclcc.fr/-sci/sor/eng_pdf/sor_thymus.pdf

Ruffié P, Gory-Delabaere G, Fervers B, Lehmann M, Regnard JF, Resbeut M (2000) *Standards, Options et Recommandations pour la prise en charge des patients atteints de tumeurs épithéliales du thymus [online]*, Fédération Nationale des Centres de Lutte Contre le Cancer (ed) November 2000. Available: URL: http://www.fnclcc.fr/-sci/sor/pdf/thymus_integrale_0300.pdf

Ruffié P, Gory-Delabaere G, Fervers B, Lehmann M, Regnard JF, Resbeut M (2000) *Standards, Options et Recommandations pour la prise en charge des patients atteints de tumeurs épithéliales du thymus [online]*, Fédération Nationale des Centres de Lutte Contre le Cancer (ed) November 2000. Available: URL: http://www.fnclcc.fr/-sci/sor/pdf/thymus_abregee_0199.pdf

Ruffie P, Gory-Delabaere G, Fervers B, Lehmann M, Regnard JF, Resbeut M (1999) Standards, options et recommandations pour la prise en charge des patients atteints de tumeurs épithéliales du thymus. *Bull Cancer* **86**: 365–384

Ruffié P, Bretel JJ, Lagrange JL, Lehmann M (1998). Standards, options et recommandations pour la prise en charge des patients atteints de tumeurs épithéliales du thymus. In *Recommandations pour la pratique clinique en cancérologie [CD-ROM]*, Fédération Nationale des Centres de Lutte Contre le Cancer (ed) 2nd edn. Paris: FNCLCC, John Libbey EUROTEXT. Standards, Options et Recommandations

### Lung cancer

#### Non-small-cell lung cancer

Brechot JM, Molina T, Theobald S, Depierre A, Lagrange JL, Astoul P, Baldeyrou P, Bardet E, Bazelly B, Breton JL, Douillard JY, Grivaux M, Jacoulet P, Khalil A, Lemarié E, Martinet Y, Massard G, Milleron B, Moro-Sibilot D, Paesmans M, Pujol H, Quoix AE, Ranfaing E, Riviere A, Sancho-Garnier H, Souquet PJ, Spaeth D, Stoebner-Delbarre A (2002) Standards, Options et Recommandations 2000 pour l'intérêt pronostique des oncogènes et génes suppresses de tumeurs dans les cancers bronchopulmonaires non à petites cellules. *Bull Cancer* **89**: 857–867

Depierre A, Lagrange JL, Théobald S, Astoul P, Baldeyrou P, Bardet E, Bazelly B, Bréchot JM, Breton JL, Douillard JY, Grivaux M, Jacoulet P, Khalil A, Lemarié E, Martinet Y, Massard G, Milleron B, Moro-Sibilot D, Paesmans M, Pujol JL, Quoix E, Ranfaing E, Rivière A, Sancho-Garnier H, Souquet PJ, Spaeth D, Stoebner-Delbarre A, Thiberville L, Touboul E, Vaylet F, Vergnon JM, Westeel V (2002) In *Cancer Bronchopulmonaire non à petite cellules*, Fédération Nationale des Centres de Lutte Contre le Cancer, Société de Pneumologie de langue Française (eds) Vol. 13. Paris: John Libbey EUROTEXT. Standards, Options et Recommandations

Depierre A, Lagrange JL, Théobald S, Astoul P, Baldeyrou P, Bardet E, Bazelly B, Bréchot JM, Breton JL, Douillard JY, Grivaux M, Jacoulet P, Khalil A, Lemarié E, Martinet Y, Massard G, Milleron B, Moro-Sibilot D, Paesmans M, Pujol JL, Quoix E, Ranfaing E, Rivière A, Sancho-Garnier H, Souquet PJ, Spaeth D, Stoebner-Delbarre A, Thiberville L, Touboul E, Vaylet F, Vergnon JM, Westeel V (2002) In *Cancer bronchopulmonaire non à petite cellules [online]*, Fédération Nationale des Centres de Lutte Contre le Cancer, Société de Pneumologie de langue Française (eds) April 2002. Available: URL: http://www.fnclcc.fr/-sci/sor/pdf/poumon_npc_integrale_0800.pdf

Depierre A, Lagrange JL, Théobald S, Astoul P, Baldeyrou P, Bardet E, Bazelly B, Bréchot JM, Breton JL, Douillard JY, Grivaux M, Jacoulet P, Khalil A, Lemarié E, Martinet Y, Massard G, Milleron B, Moro-Sibilot D, Paesmans M, Pujol JL, Quoix E, Ranfaing E, Rivière A, Sancho-Garnier H, Souquet PJ, Spaeth D, Stoebner-Delbarre A, Thiberville L, Touboul E, Vaylet F, Vergnon JM, Westeel V (2002) In *Cancer bronchopulmonaire non à petite cellules [online]*, Fédération Nationale des Centres de Lutte Contre le Cancer, Société de Pneumologie de langue Française (eds) April 2002. Available: URL: http://www.fnclcc.fr/-sci/sor/pdf/poumon_npc_arbres_0800.pdf

Depierre A, Lagrange JL, Théobald S, Astoul P, Baldeyrou P, Bardet E, Bazelly B, Bréchot JM, Breton JL, Douillard JY, Grivaux M, Jacoulet P, Khalil A, Lemarié E, Martinet Y, Massard G, Milleron B, Moro-Sibilot D, Paesmans M, Pujol JL, Quoix E, Ranfaing E, Rivière A, Sancho-Garnier H, Souquet PJ, Spaeth D, Stoebner-Delbarre A, Thiberville L, Touboul E, Vaylet F, Vergnon JM, Westeel V (2002) In *Cancer bronchopulmonaire non à petite cellules [online]*, Fédération Nationale des Centres de Lutte Contre le Cancer, Société de Pneumologie de langue Française (eds) April 2002. Available: URL: http://www.fnclcc.fr/-sci/sor/pdf/poumon_npc_abregee_0800.pdf

Bardet E, Moro-Sibilot D, Le Chevalier T, Massard G, Douillard JY, Theobald S, Astoul P, Baldeyrou P, Bazelly B, Brechot J, Breton JL, Grivaux P, Jacoulet P, Khalil A, Lemarie E, Martinet Y, Milleron B, Paesmans M, Pujol JL, Quoix AE, Ranfaing E, Riviere A, Sancho-Garnier H, Souquet PJ, Spaeth D, Stoebner-Delbarre A, Thiberville L, Touboul E, Vaylet F, Vergnon JM, Westeel V, Depierre A, Lagrange JL (2001) Standards, options et recommandations pour la prise en charge thérapeutique des patients atteints d'un cancer bronchopulmonaire primitif non à petites cellules localement avancé. *Bull Cancer* **88**: 369–387

Touboul E, Lagrange JL, Theobald S, Astoul P, Baldeyrou P, Bardet E, Bazelly B, Brechot J, Breton JL, Douillard JY, Grivaux M, Jacoulet P, Khalil A, Le Chevalier T, Lemarie E, Martinet Y, Massard G, Milleron B, Moro-Sibilot D, Paesmans M, Pujol JL, Quoix AE, Ranfaing E, Riviere A, Sancho-Garnier H, Souquet PJ, Spaeth D, Stoebner-Delbarre A, Thiberville L, Vaylet F, Vergnon JM, Westeel V, Depierre A (2001) Standards, Options et Recommandations pour la prise en charge des patients atteints d'un cancer bronchique primitif de stade I ou II traités par irradiation exclusive. *Cancer Radiother* **5**: 452–463

#### Malignant mesothelioma

Ruffié P, Lehmann M, Galateau-Salle F, Lagrange JL, Pairon JC (2001) Malignant mesothelioma. *Br J Cancer* **84** (Suppl 2): 49–50

Ruffié P, Lehmann M, Galateau-Salle F, Lagrange JL, Pairon JC (2001) SOR malignant mesothelioma [online]. *Electron J Oncol* **1**: 32–42. Available: URL: http://www.elecjoncol.org/

Galateau-Sallé F, Lagrange JL, Lehmann M, Pairon JC, Ruffié P (2000) In *Standards, Options et Recommandations pour la prise en charge des patients atteints de mésothéliome malin de la plèvre [online]*, Fédération Nationale des Centres de Lutte Contre le Cancer (ed) November 2000. Available: URL: http://www.fnclcc.fr/-sci/sor/pdf/mesotheliome_integrale_0500.pdf

Ruffié P, Lehmann M, Galateau-Sallé F, Lagrange JL, Pairon JC (2000) Standards, options et recommandations pour la prise en charge des patients atteints de mésothéliome malin de la plèvre. *Presse Med* **29**: 1432–1436

Ruffié P, Lehmann M, Galateau-Sallé F, Lagrange JL, Pairon JC (2000) In *Standards, Options et Recommandations pour la prise en charge des patients atteints de mésothéliome malin de la plèvre [online]*, Fédération Nationale des Centres de Lutte Contre le Cancer (ed) November 2000. Available: URL: http://www.fnclcc.fr/-sci/sor/pdf/mesotheliome_abregee_0599.pdf

Ruffié P, Lehmann M, Galateau-Sallé F, Lagrange JL, Pairon JC (1998) Standards, options et recommandations pour la prise en charge des patients atteints de mésothéliome malin de la plèvre. In Recommandations pour la pratique clinique en cancérologie [CD-ROM], Fédération Nationale des Centres de Lutte Contre le Cancer (ed) 2nd edn. Paris: FNCLCC, John Libbey EUROTEXT. Standards, Options et Recommandations

Ruffié P, Lehmann M, Galateau-Sallé F, Lagrange JL, Pairon JC (1998). Standards, options et recommandations pour la prise en charge des patients atteints de mésothéliome malin de la plèvre. *Bull Cancer* **85**: 545–561

### Miscellaneous

Bugat R, Bataillard A, Lesimple T, Voigt JJ, Culine S, Lortholary A, Merrouche Y, Ganem G, Kaminsky MC, Negrier S, Perol M, Laforet C, Bedossa P, Bertrand G, Coindre JM, Fizazi K (2002) Standards, Options et Recommandations 2002 pour la prise en charge des patients atteints de carcinomes de site primitif inconnu (rapport abrégé). *Bull Cancer* **89**: 869–875

## PAEDIATRIC MALIGNANCIES

### Bone and soft-tissue cancers

*Bone cancer*

### Osteosarcoma

Philip T, Blay JY, Brunat-Mentigny M, Carrie C, Chauvot P, Farsi F, Fervers B, Gentet JC, Giammarile F, Kohler R, Mathoulin S, Patricot LM, Thiesse P (2001) Osteosarcoma. *Br J Cancer* **84** (Suppl 2): 78–80

Philip T, Blay JY, Brunat-Mentigny M, Carrie C, Chauvot P, Farsi F, Fervers B, Gentet JC, Giammarile F, Kohler R, Mathoulin S, Patricot LM, Thiesse P (2001) SOR osteosarcoma [online]. *Electron J Oncol* **1**: 112–116 Available: URL: http://www.elecjoncol.org/

Philip T, Blay JY, Brunat-Mentigny M, Carrie C, Chauvot P, Farsi F, Fervers B, Gentet JC, Giammarile F, Kohler R, Mathoulin S, Patricot L, Thiesse P, Société Française d'Oncologie Pédiatrique (2000) In *Standards, Options et Recommandations pour le diagnostic, le traitement et la surveillance de l'ostéosarcome [online]*, Fédération Nationale des Centres de Lutte Contre le Cancer (ed) November 2000. Available: URL: http://www.fnclcc.fr/-sci/sor/pdf/osteosarcome_integrale_1298.pdf

Philip T, Blay JY, Brunat-Mentigny M, Carrie C, Chauvot P, Farsi F, Fervers B, Gentet JC, Giammarile F, Kohler R, Mathoulin S, Patricot L, Thiesse P (1999) Standards, options et recommandations pour le diagnostic, le traitement et la surveillance de l'ostéosarcome. *Bull Cancer* **86**: 159–176

Philip T, Blay JY, Brunat-Mentigny M, Carrie C, Chauvot P, Farsi F, Fervers B, Gentet JC, Giammarile F, Kohler R, Mathoulin S, Patricot L, Thiesse P, Fédération Nationale des Centres de Lutte Contre le Cancer, Société Française d'Oncologie Pédiatrique (1998). Standards, options et recommandations pour le diagnostic, le traitement et la surveillance de l'ostéosarcome. In *Recommandations pour la pratique clinique en cancérologie [CD-ROM]*, Fédération Nationale des Centres de Lutte Contre le Cancer (ed) 2nd edn. Paris: FNCLCC, John Libbey EUROTEXT. Standards, Options et Recommandations

Brunat-Mentigny M, Blay JY, Carrie C, Chauvot P, Fervers B, Gentet JC, Kohler R, Mathoulin S, Patricot L, Philip T, Thiesse P (1995) Standards, options et recommandations pour le diagnostic, le traitement et la surveillance de l'ostéosarcome. In *Sarcomes des tissus mous et ostéosarcomes*, Fédération Nationale des Centres de Lutte Contre le Cancer (ed) Vol. 1, pp 115–168. Paris: Arnette Blackwell. Standards, Options et Recommandations

### Ewing's sarcoma

Pinkerton CR, Bataillard A, Guillo S, Oberlin O, Fervers B, Philip T (2001) Treatment strategies for metastatic Ewing's sarcoma. *Eur J Cancer* **37**: 1338–1344

#### Rhabdomyosarcoma

Sommelet D, Pinkerton R, Brunat-Mentigny M, Farsi F, Martel I, Philip T, Ranchère-Vince D, Thiesse P, Société Française d'Oncologie Pédiatrique (2000) In *Standards, Options et Recommandations pour la prise en charge des patients atteints de rhabdomyosarcomes et autres tumeurs mesenchymateuses malignes non-rhabdomyosarcomes de l'enfant [online]*, Fédération Nationale des Centres de Lutte Contre le Cancer (ed) November 2000. Available: URL: http://www.fnclcc.fr/-sci/sor/pdf/rhabdomyosarcome_integrale_0397.pdf

Pinkerton R, Sommelet D, Brunat-Mentigny M, Farsi F, Martel I, Philip T, Ranchère-Vince D, Thiesse P, Fédération Nationale des Centres de Lutte Contre le Cancer, Société Française d'Oncologie Pédiatrique (1998) Standards, options et recommandations pour la prise en charge des patients atteints de rhabdomyosarcomes et autre tumeurs mesenchymateuses malignes non-rhabdomyosarcomes de l'enfant. *Bull Cancer* **85**: 1015–1042.

Pinkerton R, Sommelet D, Brunat-Mentigny M, Farsi F, Martel I, Philip T, Ranchère-Vince D, Thiesse P, Fédération Nationale des Centres de Lutte Contre le Cancer, Société Française d'Oncologie Pédiatrique (1998) Standards, options et recommandations pour la prise en charge des patients atteints de rhabdomyosarcomes ou de tumeurs mesenchymateuses malignes autres de l'enfant. In *Recommandations pour la pratique clinique en cancérologie [CD-ROM]*, Fédération Nationale des Centres de Lutte Contre le Cancer (ed) 2nd edn. Paris: FNCLCC, John Libbey EUROTEXT. Standards, Options et Recommandations

### Tumours of the central nervous system

*Medulloblastoma*

Alapetite C, Chastagner P, Choux M, Doz F, Gaboriaud G, Pontvert D, Sarradet A (2000) In *Standards, options et recommandations. Médulloblastome de l'enfant [online]*, Fédération Nationale des Centres de Lutte Contre le Cancer (ed) November 2000. Available: URL: http://www.fnclcc.fr/-sci/sor/pdf/medulloblastome_integrale_0397.pdf

Doz F, Alapetite C, Chastagner P, Choux M, Gaboriaud G, Pontvert D, Sarradet A, Fédération Nationale des Centres de Lutte Contre le Cancer, Société Française d'Oncologie Pédiatrique (1999) Médulloblastome de l'enfant. In *Pédiatrie II. Neuroblastome et médulloblastome*, Fédération Nationale des Centres de Lutte Contre le Cancer (ed) Vol. 10. Paris: John Libbey EUROTEXT. p. 167–222. Standards, Options et Recommandations

Doz F, Alapetite C, Chastagner P, Choux M, Gaboriaud G, Pontvert D, Sarradet A (1998) Standards, options et recommandations. Médulloblastome de l'enfant. In *Recommandations pour la pratique clinique en cancérologie [CD-ROM]*, Fédération Nationale des Centres de Lutte Contre le Cancer (ed) 2nd edn. Paris: FNCLCC, John Libbey EUROTEXT. Standards, Options et Recommandations

#### Neuroblastoma

Bergeron C, Blanc-Vincent MP, Bouvier R, Combaret V, Gauthier F, Giammarile F, Hartmann O, Mathieu P, Michon J, Neuenschwander S, Philip T, Plantaz D, Ranchère D, Rubie H, Thiesse P (2000) In *Standards, Options et Recommandations pour le neuroblastome de l'enfant [online]*, Fédération Nationale des Centres de Lutte Contre le Cancer (ed) November 2000. Available: URL: http://www.fnclcc.fr/-sci/sor/enfant/c_neuroblastome/index.htm

Bergeron C, Blanc-Vincent MP, Bouvier R, Combaret V, Gauthier F, Giammarile F, Hartmann O, Mathieu P, Michon J, Neuenschwander S, Philip T, Plantaz D, Ranchère D, Rubie H, Thiesse P (2000) *Standards, Options et Recommandations pour le neuroblastome de l'enfant [online]*, Fédération Nationale des Centres de Lutte Contre le Cancer (ed) November 2000. Available: URL: http://www.fnclcc.fr/-sci/sor/pdf/neuroblastome_integrale_0597.pdf

Pinkerton R, Blanc-Vincent MP, Bergeron C, Fervers B, Philip T (2000) Induction chemotherapy in metastatic neuroblastoma–does dose influence response? A critical review of published data. Standards, options and Recommandations (SOR) project of the National Federation of French Cancer Centres (FNCLCC). *Eur J Cancer* **36**: 1808–1815

Bergeron C, Blanc-Vincent MP, Bouvier R, Combaret V, Gauthier F, Giammarile F, Hartmann O, Mathieu P, Michon J, Neuenschwander S, Philip T, Plantaz D, Ranchère D, Rubie H, Thiesse P (1998) Standards, options et recommandations pour le neuroblastome de l'enfant. In: *Recommandations pour la pratique clinique en cancérologie [CD-ROM]*, Fédération Nationale des Centres de Lutte Contre le Cancer (ed) 2nd edn. Paris: FNCLCC, John Libbey EUROTEXT. Standards, Options et Recommandations

Bergeron C, Blanc-Vincent MP, Bouvier R, Combaret V, Gauthier F, Giammarile F, Hartmann O, Mathieu P, Michon J, Neuenschwander S, Philip T, Plantaz D, Ranchère D, Rubie H, Thiesse P, Fédération Nationale des Centres de Lutte Centre le Cancer, Société Française d'Oncologie Pédiatrique (1999) Neuroblastome de l'enfant. In *Pédiatrie II. Neuroblastome et médulloblastome*, Fédération Nationale des Centres de Lutte Contre le Cancer (ed) Vol. 10, pp 1–165. Paris: John Libbey EUROTEXT. Standards, Options et Recommandations

## TREATMENT

### Cytotoxic drugs

Crauste-Manciet S, Faure P, Latour JF, Madelaine-Chambrin I, Saulnier JL, Canal P, Cazin JL, Robert J, Husson MC, Montagnier-Pétrissans C, Centre National Hospitalier d'Information sur le Médicament (CNHIM) (1998) Médicaments anticancéreux. In *Recommandations pour la pratique clinique en cancérologie [CD-ROM]*, Fédération Nationale des Centres de Lutte Contre le Cancer (ed) 2nd edn. Paris: FNCLCC, John Libbey EUROTEXT. Standards, Options et Recommandations

Crauste-Manciet S, Faure P, Latour JF, Madelaine-Chambrin I, Saulnier JL (1995) Standards, options et recommandations pour l'utilisation pratique des anticancéreux. *Bull Cancer* **82** (Suppl 4): 319s–452s

### Erythropoietin

Spaëth D, Marchal C, Bataillard A, Blanc-Vincent MP (2000) In *Elément de la mise à jour 1999 des Standards, Options, Recommandations pour l'utilisation de l'érythropoïétine en cancérologie [online]*, Fédération Nationale des Centres de Lutte Contre le Cancer (ed) November 2000. Available: URL: http://www.fnclcc.fr/-sci/sor/pdf/erythropoietine_integrale_0599.pdf

Spaëth D, Marchal C, Bataillard A, Blanc-Vincent MP (1999) Eléments de la mise à jour 1999 des standard, options, recommandations pour l'utilisation de l'érythropoïétine en cancérologie. *Bull Cancer* **86**: 631–639

Spaëth D, Marchal C, Blanc-Vincent MP (1998) Standards, options et recommandations pour l'utilisation de l'érythropoïetine en cancérologie. *Bull Cancer* **85**: 337–346

Spaëth D, Marchal C, Blanc-Vincent MP (1998) Standards, options et recommandations pour l'utilisation de l'érythropoïetine en cancérologie. In *Recommandations pour la pratique clinique en cancérologie [CD-ROM]*, Fédération Nationale des Centres de Lutte Contre le Cancer (ed) 2nd edn. Paris: FNCLCC, John Libbey EUROTEXT. Standards, Options et Recommandations

### Haematological growth factors

Blay JY, Le Cesne A, Blanc-Vincent MP, Fervers B, Latour JF, Philip T (2001) In *Standards, Options et Recommandations pour l'utilisation des facteurs de croissance hématopoïétique en cancérologie [online]*, Fédération Nationale des Centres de Lutte Contre le Cancer (ed) September 2001. Available: URL: http://www.fnclcc.fr/-sci/sor/pdf/fch_integrale_0599.pdf

Blay JY, Le Cesne A, Blanc-Vincent MP, Fervers B, Latour JF, Philip T (2001) *Standards, Options et Recommandations pour l'utilisation des facteurs de croissance hématopoïétique en cancérologie [online]*, Fédération Nationale des Centres de Lutte Contre le Cancer (ed) April 2001. Available: URL: http://www.fnclcc.fr/-sci/sor/pdf/fch_abregee_0599.pdf

Blay JY, Le Cesne A, Blanc-Vincent MP, Fervers B, Latour JF, Philip T (2000) Standards, options et recommandations pour l'utilisation des facteurs de croissance hématopoïétique en cancérologie. *Presse Med* **29**: 2004–2008

Blay JY, Le Cesne A, Blanc-Vincent MP, Fervers B, Latour JF, Philip T (1998) Standards, options et recommandations pour l'utilisation des facteurs de croissance hématopoïétique en cancérologie. In *Recommandations pour la pratique clinique en cancérologie [CD-ROM]*, Fédération Nationale des Centres de Lutte Contre le Cancer (ed) 2nd edn. Paris: FNCLCC, John Libbey EUROTEXT. Standards, Options et Recommandations

Blay JY, Fervers B, Latour JF, Philip T (1995) Standards, options et recommandations pour l'utilisation des facteurs de croissance hématopoïétique en cancérologie. *Bull Cancer* **82** (Suppl 4): 487s–508s

### Antiemetic therapy

Rivière A, Beckendorf V, Blanc-Vincent MP, Bonneterre J, Bui NB, Catimel G, Cupissol D, Fargeot P, Hecquet B, Krakowski I, Madelaine-Chambrin I, Mihura J, Roche H, Ruffle P, Schell M, Schneider M, Philip T (2001) In *Standards, Options et Recommandations pour l'utilisation des antiémétiques en cancérologie [online]*, Fédération Nationale des Centres de Lutte Contre le Cancer (ed) October 2001. Available: URL: http://www.fnclcc.fr/-sci/sor/traitements/antiemetiques.htm

Rivière A, Beckendorf V, Blanc-Vincent MP, Bonneterre J, Bui NB, Catimel G, Cupissol D, Fargeot P, Hecquet B, Krakowski I, Madelaine-Chambrin I, Mihura J, Roche H, Ruffie P, Schell M, Schneider M, Philip T (1998) Standards, options et recommandations pour l'utilisation des antiémétiques en cancérologie. In *Recommandations pour la pratique clinique en cancérologie [CD-ROM]*, Fédération Nationale des Centres de Lutte Contre le Cancer (ed) 2nd edn. Paris: FNCLCC, John Libbey EUROTEXT. Standards, Options et Recommandations

Rivière A, Beckendorf V, Bonneterre J, Bui NB, Catimel G, Cupissol D, Fargeot P, Hecquet B, Krakowski I, Madelaine-Chambrin I, Mihura J, Roche H, Ruffie P, Schneider M, Philip T (1995) Standards, options et recommandations pour l'utilisation des antiémétiques en cancérologie. *Bull Cancer* **82** (Suppl 4): 453s–485s

### Pain management

Krakowski I, Theobald S, Balp L, Bonnefol MP, Chwetzoff G, Collard O, Collin E, Couturier M, Delorme T, Duclos R, Eschalier A, Fergane B, Larue F, Magnet M, Minello C, Navez ML, Richard A, Richard B, Rostaing-Rigattieri S, Rousselot H, Santolaria N, Torloting G, Toussaint S, Vuillemin N, Wagner JP, Fabre N (2002). Standards, Options et Recommandations 2002 pour les traitements antalgiques médicamenteux des douleurs cancéreuses par excès de nociception chez l'adulte, mise à jour (rapport abrégé). *Bull Cancer* **89**: 1067–1074

Krakowski I, Gestin Y, Jaulmes F, Lakdja F, Meynadier J, Poulain P, Pozzo di Borgo C, Rebattu P, Schach R, Goldberg J, Boureau F, Falcoff H, Guillain H, Larue F, Magnet M, Salamagne M, Serrie A, Trechot P, Verdie JC (2001) In *Recommandations pour une bonne pratique dans la douleur du cancer chez l'adulte et l'enfant [online]*, Fédération Nationale des Centres de Lutte Contre le Cancer (ed) June 2001. Available: URL: http://www.fnclcc.fr/-sci/sor/pdf/douleur_integrale_0897.pdf

Krakowski I, Gestin Y, Jaulmes F, Lakdja F, Meynadier J, Poulain P, Pozzo di Borgo C, Rebattu P, Schach R, Goldberg J, Boureau F, Falcoff H, Guillain H, Larue F, Magnet M, Salamagne M, Serrie A, Trechot P, Verdie JC (2001) In *Recommandations pour une bonne pratique dans la douleur du cancer chez l'adulte et l'enfant [online]*, Fédération Nationale des Centres de Lutte Contre le Cancer (ed) June 2001. Available: URL: http://www.fnclcc.fr/-sci/sor/pdf/douleur_arbres_0897.pdf

Krakowski I, Boureau F, Falcoff H, Gestin Y, Goldberg J, Guillain H, Jaulmes F, Lakdja F, Larue F, Magnet M, Meynadier J, Poulain P, Pozzo di Borgo C, Rebattu P, Salamagne M, Serrie A, Schach R, Trechot P, Verdie JC, Fédération Nationale des Centres de Lutte Contre le Cancer, Société Francophone d'Etude de la Douleur (1998). Recommandations pour une bonne pratique dans la prise en charge de la douleur du cancer chez l'adulte et l'enfant. In *Recommandations pour la pratique clinique en cancérologie [CD-ROM]*, Fédération Nationale des Centres de Lutte Contre le Cancer (ed) 2nd edn. Paris: FNCLCC, John Libbey EUROTEXT. Standards, Options et Recommandations

Krakowski I, Falcoff H, Gestin Y, Goldberg J, Guillain H, Jaulmes F, Lakdja F, Larue F, Magnet M, Meynadier J, Poulain P, Pozzo di Borgo C, Rebattu P, Salamagne M, Serrie A, Schach R, Trechot P, Verdie JC (1996) Recommandations pour une bonne pratique dans la prise en charge de la douleur du cancer chez l'adulte et l'enfant. *Bull Cancer* **83** (Suppl 1): 9s–79s

Krakowski I, Falcoff H, Gestin Y, Goldberd Y, Guillain H, Jaulmes F, Lakdja F, Larue F, Magnet M, Meynadier J, Poulain P, Pozzo di Borgo C, Rebattu P, Salamagen M, Serrie A, Schach R, Trechot P, Verdie JC (1995) Recommandations pour une bonne pratique dans la prise en charge de la douleur chez l'adulte et l'enfant. *Bull Cancer* **82** (Suppl 4): 245s–315s

### Nutritional support in haemato-oncology

Raynard B, Nitenberg G, Gory-Delabaere G, Bourhis JH, Bachmann P, Bensadoun RJ, Desport JC, Kere D, Schneider S, Senesse P, Bordigoni P, Dieu L (2002) Standards, Options et Recommandations pour la nutrition artificielle au cours et au decours de la greffe de cellules souches hématopoïétiques (CSH). *Bull Cancer* **89**: 381–400

Raynard B, Nitenberg G, Gory-Delabaere G, Bourhis JH, Bachmann P, Bensadoun RJ, Desport JC, Kere D, Schneider S, Senesse P, Bordigoni P, Dieu L (2002) *Standards, Options et Recommandations 2002 pour la nutrition artificielle au cours et au décours de la greffe de cellules souches hématopoïétiques (CSH) [online]*, Fédération Nationale des Centres de Lutte Contre le Cancer (ed) July 2002. Available: URL: http://www.fnclcc.fr/-sci/sor/pdf/nutrition_greffe_integ ral_0202.pdf

Raynard B, Nitenberg G, Gory-Delabaere G, Bourhis JH, Bachmann P, Bensadoun RJ, Desport JC, Kere D, Schneider S, Senesse P, Bordigoni P, Dieu L (2002) In *Standards, Options et Recommandations 2002 pour la nutrition artificielle au cours et au décours de la greffe de cellules souches hématopoïétiques (CSH) [online]*, Fédération Nationale des Centres de Lutte Contre le Cancer (ed) July 2002. Available: URL: http://www.fnclcc.fr/-sci/sor/pdf/nutrition_greffe_arbres_0202.pdf

Raynard B, Nitenberg G, Gory-Delabaere G, Bourhis JH, Bachmann P, Bensadoun RJ, Desport JC, Kere D, Schneider S, Senesse P, Bordigoni P, Dieu L (2002) In *Standards, Options et Recommandations 2002 pour la nutrition artificielle au cours et au décours de la greffe de cellules souches hématopoïétiques (CSH) [online]*, Fédération Nationale des Centres de Lutte Contre le Cancer (ed) July 2002. Available: URL: http://www.fnclcc.fr/-sci/sor/pdf/nutrition_greffe_abrege_0202.pdf

Bachmann P, Marti-Massoud C, Blanc-Vincent MP, Desport JC, Colomb V, Dieu L, Kere D, Melchior J, Nitenberg G, Raynard B, Roux-Bournay P, Schneider S, Senesse P (2002) *Standards, Options et Recommandations: nutrition en situation palliative ou terminale de l'adulte porteur de cancer évolutif [online]*, Fédération Nationale des Centres de Lutte Contre le Cancer (ed) January 2002. Available: URL: http://www.fnclcc.fr/-sci/sor/pdf/nutrition_palliative_integrale_0701.pdf

Bachmann P, Marti-Massoud C, Blanc-Vincent MP, Desport JC, Colomb V, Dieu L, Kere D, Melchior J, Nitenberg G, Raynard B, Roux-Bournay P, Schneider S, Senesse P (2002) In *Standards, Options et Recommandations: nutrition en situation palliative ou terminale de l'adulte porteur de cancer évolutif [online]*, Fédération Nationale des Centres de Lutte Contre le Cancer (ed) January 2002. Available: URL: http://www.fnclcc.fr/-sci/sor/pdf/nutrition_palliative_abregee_0701.pdf

Bachmann P, Marti-Massoud C, Blanc-Vincent MP, Desport JC, Colomb V, Dieu L, Kere D, Melchior J, Nitenberg G, Raynard B, Roux-Bournay P, Schneider S, Senesse P (2001) Standards, Options, Recommandations: nutrition en situation palliative ou terminale de l'adulte porteur de cancer évolutif. *Bull Cancer* **88**: 985–1006

Schneider S, Blanc-Vincent MP, Nitenberg G, Senesse P, Bachmann P, Colomb V, Desport JC, Gory-Delabaere G, Kere D, Raynard B, Melchior JC (2001) Standards, Options et Recommandations: Nutrition artificielle à domicile du malade cancéreux adulte. *Bull Cancer* **88**: 605–618

Schneider S, Blanc-Vincent MP, Nitenberg G, Senesse P, Bachmann P, Colomb V, Desport JC, Gory-Delabaere G, Kere D, Raynard B, Melchior JC (2001) In *Standards, Options et Recommandations: Nutrition artificielle à domicile du malade cancéreux adulte [online]*, Fédération Nationale des Centres de Lutte Contre le Cancer (ed) July 2001. Available: URL: http://www.fnclcc.fr/-sci/sor/pdf/nutrition_domicile_integrale_0301.pdf

Desport JC, Blanc-Vincent MP, Gory-Delabaere G, Bachmann P, Béal J, Benamouzig R, Colomb V, Kere D, Melchior JC, Nitenberg G, Raynard B, Schneider S, Senesse P (2000) Standards, options et recommandations (SOR) pour l'utilisation des médicaments orexigènes en cancérologie. *Bull Cancer* **87**: 315–328

Desport JC, Blanc-Vincent MP, Gory-Delabaere G, Bachmann P, Béal J, Benamouzig R, Colomb V, Kere D, Melchior JC, Nitenberg G, Raynard B, Schneider S, Senesse P (2000) In *Standards, Options et Recommandations pour l'utilisation des médicaments orexigènes en cancérologie [online]*, Fédération Nationale des Centres de Lutte Contre le Cancer (ed) November 2000. Available: URL: http://www.fnclcc.fr/-sci/sor/pdf/orexigene_integrale_0100.pdf

Nitenberg G, Blanc-Vincent MP, Philip T (2000) Standards, options et recommandations (SOR): assistance nutritionnelle en onco-hématologie. *Bull Cancer* **87**: 311–313

### Complimentary medicine

Schraub S (1998) Standards, options et recommandations concernant les médecines paralléles. In Recommandations pour la pratique clinique en cancérologie [CD-ROM], Fédération Nationale des Centres de Lutte Contre le Cancer (ed) 2nd edn. Paris: FNCLCC, John Libbey EUROTEXT. Standards, Options et Recommandations

## GOOD PRACTICE IN ONCOLOGY

### Infection and cancer

*General principles*

Viot M, Blanc-Vincent MP, Béal J, Biron P, Boutard P, Bussy Malgrange V, Crokaert F, Escande MC, Fuhrman C, Lesimple T, Pény J, Pottecher B, Raveneau J, Senet JM, Thyss A (2000) Infection et cancer: notions générales et questions actuelles. *Presse Med* **29**: 1630–1633

Viot M, Béal J, Biron P, Blanc-Vincent MP, Boutard P, Bussy V, Crokaert F, Escande MC, Fuhrman C, Lesimple T, Pény J, Pottecher B, Raveneau J, Senet JM, Thyss A (1999) Infection et cancer: notions générales et questions actuelles. In *Infection et cancer*, Fédération Nationale des Centres de Lutte Contre le Cancer (ed) Vol. 8, pp 9–36. Paris: John Libbey EUROTEXT. Standards, Options et Recommandations

#### Good practice in blood culture

Bussy Malgrange V, Blanc-Vincent MP, Escande MC, Fuhrman C, Béal J, Boineau F, Biron P, Crokaert F, Lesimple T, Pottecher B, Raveneau J, Senet JM, Viot M (2000) Standards, Options et Recommandations pour une bonne pratique des hémocultures en cancérologie. *Presse Med* **29**: 1532–1534

Bussy V, Escande MC, Fuhrman C, Boineau F, Blanc-Vincent MP, Béal J, Biron P, Crokaert F, Lesimple T, Pottecher B, Raveneau J, Senet JM, Viot M (1999) Standards, options et recommandations pour une bonne pratique des hémocultures en cancérologie. In *Infection et cancer*, Fédération Nationale des Centres de Lutte Contre le Cancer (ed) Vol. 8, pp 37–62. Paris: John Libbey EUROTEXT. Standards, Options et Recommandations

Bussy V, Escande MC, Fuhrman C, Boineau F, Blanc-Vincent MP, Béal J, Biron P, Crokaert F, Lesimple T, Pottecher B, Raveneau J, Senet JM, Viot M (1998) Standards, options et recommandations pour une bonne pratique des hémocultures en cancérologie. In *Recommandations pour la pratique clinique en cancérologie [CD-ROM]*, Fédération Nationale des Centres de Lutte Contre le Cancer (ed) 2nd edn. Paris: FNCLCC, John Libbey EUROTEXT. Standards, Options et Recommandations

#### Candidiasis

Senet JM, Herbrecht R, Blanc-Vincent MP, Béal J, Biron P, Bussy V, Crokaert F, Lesimple T, Escande MC, Fuhrman C, Pottecher B, Raveneau J, Viot M (1999) Standards, options et recommandations pour la prévention, le diagnostic et le traitement des candidoses en cancérologie. In *Infection et cancer*, Fédération Nationale des Centres de Lutte Contre le Cancer (ed) Vol. 8, pp 181–236. Paris: John Libbey EUROTEXT. Standards, Options et Recommandations

Senet JM, Herbrecht R, Blanc-Vincent MP, Béal J, Biron P, Bussy V, Crokaert F, Lesimple T, Escande MC, Fuhrman C, Pottecher B, Raveneau J, Viot M (1998) Standards, options et recommandations pour la prévention, le diagnostic et le traitement des candidoses en cancérologie. In *Recommandations pour la pratique clinique en cancérologie [CD-ROM]*, Fédération Nationale des Centres de Lutte Contre le Cancer (ed) 2nd edn. Paris: FNCLCC, John Libbey EUROTEXT. Standards, Options et Recommandations

#### Infectious complications associated with the administration of interleukin-2

Escande MC, Blanc-Vincent MP, Lesimple T, Bussy V, Béal J, Crokaert F, Biron P, Viot M, Fuhrman C, Pottecher B, Senet JM, Raveneau J (1999) Prévention, diagnostic et traitement des complications infectieuses liées á l'administration intraveineuse d'interleukine 2. In *Infection et cancer*, Fédération Nationale des Centres de Lutte Contre le Cancer (ed) Vol. 8, pp 237–260. Paris: John Libbey EUROTEXT. Standards, Options et Recommandations

Escande MC, Blanc-Vincent MP, Lesimple T, Bussy V, Béal J, Crokaert F, Biron P, Viot M, Fuhrman C, Pottecher B, Senet JM, Raveneau J (1998) Prévention, diagnostic et traitement des complications infectieuses liées á l'administration intraveineuse d'interleukine 2. In *Recommandations pour la pratique clinique en cancérologie [CD-ROM]*, Fédération Nationale des Centres de Lutte Contre le Cancer (ed) 2nd edn. Paris: FNCLCC, John Libbey EUROTEXT. Standards, Options et Recommandations

#### Infections associated with in-dwelling lines

Lesimple T, Béal J, Escande MC, Lion C, Blanc-Vincent MP, Bussy V, Biron P, Pottecher B, Crokaert F, Senet JM, Fuhrman C, Raveneau J, Viot M (1999) Standards, options et recommandations pour la prévention, le diagnostic et le traitement des infections liées aux voies veineuses en cancérologie. In *Infection et cancer*, Fédération Nationals des Centres de Lutte Contre le Cancer (ed) Vol. 8, pp 63–128. Paris: John Libbey EUROTEXT. Standards, Options et Recommandations

Lesimple T, Béal J, Escande MC, Lion C, Blanc-Vincent MP, Bussy V, Biron P, Pottecher B, Crokaert F, Senet JM, Fuhrman C, Raveneau J, Viot M (1998) Standards, options et recommandations pour la prévention, le diagnostic et le traitement des infections liées aux voies veineuses en cancérologie. In *Recommandations pour la pratique clinique en cancérologie [CD-ROM]*, Fédération Nationale des Centres de Lutte Contre le Cancer (ed) 2nd edn. Paris: FNCLCC, John Libbey EUROTEXT. Standards, Options et Recommandations

#### Nosocomial infections

Pottecher B, Herbrecht R, Blanc-Vincent MP, Bussy Malgrange V, Escande MC, Fuhrmann C, Crokaert F, Gory-Delabaere G, Senet JM, Lesimple T, Raveneau J, Béal J, Biron P, Viot M (2000) Standards, Options et Recommandations (SOR) pour la surveillance et la prévention des infections nosocomiales en cancérologie. *Bull Cancer* **87**: 557–591

Pottecher B, Herbrecht R, Blanc-Vincent MP (2000) In Standards, Options et Recommandations pour la surveillance et la prévention des infections nosocomiales en cancérologie [online], Fédération Nationale des Centres de Lutte Contre le Cancer (ed) November 2000. Available: URL: http://www.fnclcc.fr/-sci/sor/pdf/infections_nosocomiales_integrale_1199.pdf

#### Management of short duration neutropenia

Biron P, Fuhrman C, Escande MC, Blanc-Vincent MP, Crokaert F, Béal J, Bussy V, Lesimple T, Pottecher B, Raveneau J, Senet JM, Viot M (1999) Standards, options et recommandations pour la prise en charge des neutropénies courtes. In *Infection et cancer*, Fédération Nationale des Centres de Lutte Contre le Cancer (ed) Vol. 8, pp 129–179. Paris: John Libbey EUROTEXT. Standards, Options et Recommandations

Biron P, Fuhrmann C, Escande MC, Blanc-Vincent MP, Crokaert F, Béal J, Bussy V, Lesimple T, Pottecher B, Raveneau J, Senet JM, Viot M (1998) Standards, options et recommandations pour la prise en charge des neutropénies courtes. *Bull Cancer* **85**: 695–711

Biron P, Fuhrman C, Escande MC, Blanc-Vincent MP, Crokaert F, Béal J, Bussy V, Lesimple T, Pottecher B, Raveneau J, Senet JM, Viot M (1998) Standards, options et recommandations pour la prise en charge des neutropénies courtes. In Recommandations pour la pratique clinique en cancérologie [CD-ROM], Fédération Nationale des Centres de Lutte Contre le Cancer (ed) 2nd edn. Paris: FNCLCC, John Libbey EUROTEXT. Standards, Options et Recommandations

### Good practice in pathology

Denoux Y, Blanc-Vincent MP, Simony-Lafontaine J, Verriele-Beurrier V, Briffod M, Voigt JJ (2002) Standards, Options et Recommandations: bonne pratique de l'acheminement et de la prise en charge initiale d'un prélèvement en anatomie et cytologie pathologiques en cancérologie. *Bull Cancer* **89**: 401–410

Denoux Y, Blanc-Vincent MP, Simony-Lafontaine J, Verriele-Beurrier V, Briffod M, Voigt JJ (2002) In *Standards, Options et Recommandations: bonne pratique de l'acheminement et de la prise en charge initiate d'un prélèvement en anatomie et cytologie pathologiques en cancérologie [online]*, Fédération Nationale des Centres de Lutte Contre le Cancer (ed) juillet 2002. Available: URL: http://www.fnclcc.fr/-sci/sor/pdf/acheminement_anapath_integral_0202.pdf

Denoux Y, Blanc-Vincent MP, Simony-Lafontaine J, Verriele-Beurrier V, Briffod M, Voigt JJ (2002) In *Standards, Options et Recommandations: bonne pratique de l'acheminement et de la prise en charge initiate d'un prélèvement en anatomie et cytologie pathologiques en cancérologie [online]*, Fédération Nationale des Centres de Lutte Contre le Cancer (ed) July 2002. Available: URL: http://www.fnclcc.fr/-sci/sor/pdf/acheminement_anapath_arbres_0202.pdf

Coindre JM, Blanc-Vincent MP, Collin F, Mac Grogan G, Balaton A, Voigt JJ, Arnould L, Bailly C, Brifford M, Bibeau F, Fontanière B, Ghnassia JP, Guinebretière JM, Le Doussal V, Mauriac L, Merrouche Y, Sabourin JC, Sastre-Garau X, Sigal-Zafrani B, Verriele-Beurrier V, Vielh P (2001) Standards, Options et Recommandations: conduite à tenir devant une lésion de diagnostic anatomo-cyto-pathologique difficile en cancérologie. *Bull Cancer* **88**: 765–773

Coindre JM, Blanc-Vincent MP, Collin F, Mac Grogan G, Balaton A, Voigt JJ (2001) In *Standards, Options et Recommandations: conduite à tenir devant une lésion de diagnostic anatomo-cyto-pathologique difficile en cancérologie [online]*, Fédération Nationale des Centres de Lutte Contre le Cancer (ed) October 2001. Available: URL: http://www.fnclcc.fr/-sci/sor/pdf/lesion_difficile_integrale_0201.pdf

Arnould L, Fiche M, Blanc-Vincent MP, Le Doussal V, Zafrani B, Gory-Delabaere G, Brifford M, Vielh P, Voigt JJ (2000) Standards, options et recommandations pour la rédaction d'un compte-rendu d'anatomie et cytologie pathologiques en cancérologie. *Bull Cancer* **87**: 159–171.

Arnould L, Fiche M, Blanc-Vincent MP, Le Doussal V, Zafrani B, Brifford M, Vielh P, Voigt JJ, Gory-Delabaere G (2000) Standards, Options et Recommandations pour la rédaction d'un compte-rendu d'anatomie et cytologie pathologiques en cancérologie [online], Fédération Nationale des Centres de Lutte Contre le Cancer (ed) February 2000. Available: URL: http://www.fnclcc.fr/-sci/sor/pdf/cr_anapath_integrale_1099.pdf

Voigt JJ, Philip T (2000) Standards, options et recommandations (SOR): anatomie et cytologie pathologiques en cancérologie. *Bull Cancer* **87**: 155–157

### Good practice in tumour markers

Basuyau JP, Blanc-Vincent MP, Bidart JM, Daver A, Deneux L, Eche N, Gory-Delabaere G, Pichon MF, Riedinger JM (2002) In *Standards, Options et Recommandations: marqueurs tumoraux sériques du cancer du sein [online]*, Fédération Nationale des Centres de Lutte Contre le Cancer (ed) January 2002. Available: URL: http://www.fnclcc.fr/-sci/sor/pdf/sein_oncobiologie_abregee_0400.pdf

Eche N, Pichon MF, Quillien V, Gory-Delabaere G, Riedinger JM, Basuyau JP, Daver A, Blanc-Vincent MP, Buecher B, Conroy T, Bidart JM, Deneux L (2002) In *Standards, options et recommandations: marqueurs tumoraux sériques dans les cancers du côlon [online]*, Fédération Nationale des Centres de Lutte Contre le Cancer (ed) January 2002. Available: URL: http://www.fnclcc.fr/-sci/sor/pdf/oncobiologie_colon_abregee_0601.pdf

Eche N, Pichon MF, Quillien V, Gory-Delabaere G, Riedinger JM, Basuyau JP, Daver A, Blanc-Vincent MP, Buecher B, Conroy T, Bidart JM, Deneux L (2001) Standards, options et recommandations: marqueurs tumoraux sériques dans les cancers du côlon. *Bull Cancer* **88**: 1177–1206.

Eche N, Pichon MF, Quillien V, Gory-Delabaere G, Riedinger JM, Basuyau JP, Daver A, Buecher B, Conroy T, Dieu L, Deneux L (2002) Standards, options et recommandations: marqueurs tumoraux sériques dans les cancers du colon [online], Fédération Nationale des Centres de Lutte Contre le Cancer (ed) January 2002. Available: URL: http://www.fnclcc.fr/-sci/sor/pdf/oncobiologie_colon_integrale_0601.pdf

Pichon MF, Gory-Delabaere G, Basuyau JP, Eche N, Daver A, Blanc-Vincent MP, Riedinger JM, Deneux L, Bidart JM (2001) Standards, Options et Recommandations: marqueurs tumoraux sériques des cancers de la thyroïde. *Bull Cancer* **88**: 775–792

Pichon MF, Basuyau JP, Gory-Delabaere G, Eche N, Daver A, Blanc-Vincent MP, Riedinger JM, Deneux L, Bidart JM (2001) *Standards, Options et Recommandations: marqueurs tumoraux sériques des cancers de la thyroïde [online]*, Fédération Nationale des Centres de Lutte Contre le Cancer (ed) October 2001. Available: URL: http://www.fnclcc.fr/-sci/sor/pdf/oncobiologie_thyroide_integrale_0201.pdf

Pichon MF, Basuyau JP, Gory-Delabaere G, Eche N, Daver A, Blanc-Vincent MP, Riedinger JM, Deneux L, Bidart JM (2001) *Standards, Options et Recommandations: marqueurs tumoraux sériques des cancers de la thyroïde [online]*, Fédération Nationale des Centres de Lutte Contre le Cancer (ed) October 2001. Available: URL: http://www.fnclcc.fr/-sci/sor/pdf/oncobiologie_thyroide_abregee_0201.pdf

Basuyau JP, Blanc-Vincent MP, Bidart JM, Daver A, Deneux L, Eche N, Gory-Delabaere G, Pichon MF, Riedinger JM (2000) Standards, Options et Recommandations: marqueurs tumoraux sériques et cancer du sein. *Bull Cancer* **87**: 723–737

Basuyau JP, Blanc-Vincent MP, Bidart JM, Daver A, Deneux L, Eche N, Gory-Delabaere G, Pichon MF, Riedinger JM (2000) *Standards, Options et Recommandations: marqueurs tumoraux sériques et cancer du sein [online]*, Fédération Nationale des Centres de Lutte Contre le Cancer (ed) November 2000. Available: URL: http://www.fnclcc.fr/-sci/sor/pdf/oncobiologie_integrale_0400.pdf

### Good practice in chemotherapy

Heron JF, Fervers B, Latour JF, Sommelet D, Chauvergne J, Philip T (1998) Standards, options et recommandations pour une bonne pratique en chimiothérapie. In Recommandations pour la pratique clinique en cancérologie [CD-ROM], Fédération Nationale des Centres de Lutte Contre le cancer (ed) 2nd edn. Paris: FNCLCC, John Libbey EUROTEXT. Standards, Options et Recommandations

Heron JF, Fervers B, Latour JF, Sommelet D, Chauvergne J, Philip T (1995) Standards, options et recommandations pour une bonne pratique en chimiothérapie. *Bull Cancer* **82**: 823–834

### Good practice in radiotherapy

Bey P, Gaboriaud G, Peiffert D, Aletti P, Cosset JM (1998) Standards, options et recommandations pour une bonne pratique en radiothérapie externe et en curiethérapie en cancérologie. In *Recommandations pour la pratique clinique en cancérologie [CD-ROM]*, Fédération Nationale des Centres de Lutte Contre le Cancer (ed) 2nd edn. Paris: FNCLCC, John Libbey EUROTEXT. Standards, Options et Recommandations

Bey P, Gaboriaud G, Peiffert D, Aletti P, Cosset JM (1995) Standards, options et recommandations pour une bonne pratique en radiothérapie externe et en curiethérapie en cancérologie. *Bull Cancer* **82**: 811–822

### Good practice in surgery

Dauplat J, Depadt G, Abbes M, Bobin JY, Carolus JM, Chardot C, Durand JC, Fondrinier E, Fraisse J, Guillemin F, Janser JC, Julien JP, Lallement M, Lasser P, Laurent JC, Lorimier G, Michel G, Pujol H, Rauch P, Rouanet P, Saint-Aubert B, Stockle E, Verhaeghe JL (1998) Standards, options et recommandations pour une bonne pratique en chirurgie cancérologique. In *Recommandations pour la pratique clinique en cancérologie [CD-ROM]*, Fédération Nationale des Centres de Lutte Contre le Cancer, (ed) 2nd edn. Paris: FNCLCC, John Libbey EUROTEXT. Standards, Options et Recommandations

Dauplat J, Depadt G, Abbes M, Bobin JY, Carolus JM, Chardot C, Durand JC, Fondrinier E, Fraisse J, Guillemin F, Janser JC, Julien JP, Lallement M, Lasser P, Laurent JC, Lorimier G, Michel G, Pujol H, Rauch P, Rouanet P, Saint-Aubert B, Stockle E, Verhaeghe JL (1995) Standards, options et recommandations pour une bonne pratique en chirurgie cancérologique. *Bull Cancer* **82**: 795–810

### Good practice in multidisciplinary care

Bey P, Abbatucci JS, Chardot C, Farsi F, Fervers B, Philip T (1998) Standards, options et recommandations pour une bonne pratique dans l'organisation pluridisciplinaire de la cancérologie. In *Recommandations pour la pratique clinique en cancérologie [CD-ROM]*, Fédération Nationale des Centres de Lutte Contre le Cancer (ed) 2nd edn. Paris: FNCLCC, John Libbey EUROTEXT. Standards, Options et Recommandations

Chardot C, Fervers B, Bey P, Abbatucci JS, Philip T (1995) Standards, options et recommandations pour une bonne pratique dans l'organisation pluridisciplinaire de la cancérologie. *Bull Cancer* **82**(10): 780–794.

### Good practice in nuclear medicine

Maublant J, Fervers B, Herry JY, Lumbroso JD, Parmentier C, Pasquier J, Sulman C (1998) Standards, options et recommandations pour une bonne pratique de la médecine nucléaire *in vivo* en cancérologie. In *Recommandations pour la pratique clinique en cancérologie [CD-ROM]*, Fédération Nationale des Centres de Lutte Contre le Cancer (ed) 2nd edn. Paris: FNCLCC, John Libbey EUROTEXT. Standards, Options et Recommandations

Maublant J, Fervers B, Herry JY, Lumbroso JD, Parmentier C, Pasquier J, Sulman C (1998) Standards, options et recommandations pour une bonne pratique de la médecine nucléaire *in vivo* en cancérologie. *Rev ACOMEN* **4**: 63–76

### Good practice in dental care

Maire F, Borowski B, Collangettes D, Farsi F, Guichard M, Gourmet R, Kreher P (2000) In *Standards, Options et Recommandations pour une bonne pratique odontologique en cancérologie [online]*, Fédération Nationale des Centres de Lutte Contre le Cancer (ed) November 2000. Available: URL: http://www.fnclcc.fr/-sci/sor/pdf/odontologie_integrale_0699.pdf

Maire F, Borowski B, Collangettes D, Farsi F, Guichard M, Gourmet R, Kreher P (1999) Standards, Options et Recommandations pour une bonne pratique odontologique en cancérologie. *Bull Cancer* **86**: 640–665

### Good practice in psycho-oncology

Saltel P, de Raucourt D, Derzelle M, Desclaux B, Fresco R, Gauvain Piquard A, Genot JY, Landry N, Lanzarotti C, Lehmann A, Machavoine JL, Marx G, Oppenheim D, Salimpour A (1998) Standards, options et recommandations pour une bonne pratique en psycho-oncologie. In: Recommandations pour la pratique clinique en cancérologie [CD-ROM]. Fédération Nationale des Centres de Lutte Contre le Cancer (ed) 2nd edn. Paris: FNCLCC, John Libbey EUROTEXT. Standards, Options et Recommandations

Saltel P, de Raucourt D, Derzelle M, Desclaux B, Fresco R, Gauvain Piquard A, Genot JY, Landry N, Lanzarotti C, Lehmann A, Machavoine JL, Marx G, Oppenheim D, Salimpour A (1995) Standards, options et recommandations pour une bonne pratique en psycho-oncologie. *Bull Cancer* **82**: 847–864

### Good practice in imaging

Stines J, Bey P, Bertrand AF, Cambier L, Carsin A, Collay R, Delignette A, Dilhuydy MH, Hagay C, Humeau F, Kaemmerlen P, Masselot J, Netter E, Plantet MM, Sigal R, Tardivon A, Thiesse P, Tournemaine N, Troufleau P, Vanel D (1998) Standards, options et recommandations pour une bonne pratique de l'imagerie radiologique en cancérologie. In Recommandations pour la pratique clinique en cancérologie [CD-ROM]. Fédération Nationale des Centres de Lutte Contre le Cancer (ed) 2nd edn. Paris: FNCLCC, John Libbey EUROTEXT. Standards, Options et Recommandations

Stines J, Bertrand AF, Cambier L, Carsin A, Collay R, Delignette A, Dilhuydy MH, Hagay C, Humeau F, Kaemmerlen P, Masselot J, Netter E, Plantet MM, Sigal R, Tardivon A, Thiesse P, Tournemaine N, Troufleau P, Vanel D (1995) Standards, options et recommandations pour une bonne pratique de l'imagerie radiologique en cancérologie. *Bull Cancer* **82**: 835–845

### Health technology assessment

Bourguet P, Blanc-Vincent MP, Boneu A, Chauffert B, Corone C, Courbon F, Devillers A, Foehrenbach H, Lumbroso JD, Mazselin P, Montravers F, Moretti JL, Talbot JN, Avril MF, Banal A, Bey-Boeglin M, Bonichon F, Bugat R, Bui BN, Cherel P, Chevreau C, Dieu L, Fervers B, Floiras JL, Fournier R, Goldwasser F, Goupil A, Grahek D, Guillo S, Helal OB, Houlgatte A, Lacau St Guily J, Lamy de la Chapelle T, Pecking A, Périé S, Reboul F, Théodore C, Tilly H, Vaylet F, Véra P (2003) Standards, Options et Recommandations 2002 pour l'utilisation de la tomographie par émission de positons au [18F]-FDG (TEP-FDG) en cancérologie (rapport intégral). *Bull Cancer* **90**: S1–S109

Bourguet P, Blanc-Vincent MP, Boneu A, Chauffert B, Corone C, Courbon F, Devillers A, Foehrenbach H, Lumbroso JD, Mazselin P, Montravers F, Moretti JL, Talbot JN, Avril MF, Banal A, Bey-Boeglin M, Bonichon F, Bugat R, Bui BN, Cherel P, Chevreau C, Dieu L, Fervers B, Floiras JL, Fournier R, Goldwasser F, Goupil A, Grahek D, Guillo S, Helal OB, Houlgatte A, Lacau St Guily J, Lamy de la Chapelle T, Pecking A, Périé S, Reboul F, Théodore C, Tilly H, Vaylet F, Véra P (2002) Standards, Options et Recommandations pour l'utilisation de la tomographie par émission de positons au [18F]-FDG (TEP-FDG) en cancérologie [online], Fédération Nationale des Centres de Lutte Contre le Cancer (ed) March 2002. Available: URL: http://www.fnclcc.fr/-sci/sor/pdf/tep_fdg_integrale_0202.pdf

Bourguet P, Blanc-Vincent MP, Boneu A, Chauffert B, Corone C, Courbon F, Devillers A, Foehrenbach H, Lumbroso JD, Mazselin P, Montravers F, Moretti JL, Talbot JN, Avril MF, Banal A, Bey-Boeglin M, Bonichon F, Bugat R, Bui BN, Cherel P, Chevreau C, Dieu L, Fervers B, Floiras JL, Fournier R, Goldwasser F, Goupil A, Grahek D, Guillo S, Helal OB, Houlgatte A, Lacau St Guily J, Lamy de la Chapelle T, Pecking A, Périé S, Reboul F, Théodore C, Tilly H, Vaylet F, Véra P (2002) Standards, Options et Recommandations pour l'utilisation de la tomographie par émission de positons au [18F]-FDG (TEP-FDG) en cancérologie [online], Fédération Nationale des Centres de Lutte Contre le Cancer (ed) March 2002. Available: URL: http://www.fnclcc.fr/-sci/sor/pdf/tep_fdg_abregee_0202.pdf

### Good practice in dietetics

*Hospital catering*

Dayot F, Bataillard A, Keré C, Ducès F (2001) Standards, Options et Recommandations: Bonnes pratiques de diététiques en cancérologie: la prestation alimentaire [online], Fédération Nationale des Centres de Lutte Contre le Cancer (ed) October 2001. Available: URL: http://www.fnclcc.fr/pdf/dietetique_prestation_integrale_0900.pdf

Dayot F, Bataillard A, Keré C, Ducès F, Bachmann P, Blanc-Vincent MP, Besnard B, Bonneteau C, Champetier S, Claude M, Combret D, Cometto F, Duguet A, Duval N, Fink C, Freby-Lehner A, Garabige V, Lallemand Y, Massoud C, Meuric J, Montane C, Poirée B, Puel S, Rossignol G, Roux-Bournay P, Simon M, Tran M (2001) Standards, Options et Recommandations: Bonnes pratiques de diététiques en cancérologie: la prestation alimentaire. *Bull Cancer* **88**: 1007–1018

#### Nutrition for patients with cancers of the upper aerodigestive tract

Meuric J, Garabige V, Blanc-Vincent MP, Lallemand Y, Bachmann P (2002) Bonnes pratiques pour la prise en charge diététique des patients atteints de cancer des voies aérodigestives supérieures. *Nut Clin Métab* **16**: 164–184.

Meuric J, Garabige V, Blanc-Vincent MP, Lallemand Y, Bachmann P (2000) In *Bonnes pratiques pour la prise en charge diététique des patients atteints de cancer des voies aérodigestives supérieures [online]*, Fédération Nationale des Centres de Lutte Contre le Cancer (ed) November 2000. Available: URL: http://www.fnclcc.fr/-sci/sor/pdf/dietetique_vads_integrale_0399.pdf

Meuric J, Garabige V, Blanc-Vincent MP, Lallemand Y, Bachmann P (1999) Bonnes pratiques pour la prise en charge diététique des patients atteints de cancer des voies aérodigestives supérieures. *Bull Cancer* **86**: 843–854.

#### Nutrition and nutritional evaluation

Duguet A, Bachmann P, Lallemand Y, Blanc-Vincent MP, Bataillard A, Besnard B, Bonneteau C, Champetier S, Claude M, Combret D, Cometto F, Dayot F, Duval N, Fink C, Freby-Lehner A, Garabige V, Kalsh M, Massoud C, Meuric J, Montane C, Poirée B, Puel S, Rossignol G, Roux-Bournay P, Simon M, Tran M (2002) Bonnes pratiques diététiques en cancérologie: dénutrition et évaluation nutritionnelle. *Nut Clin Métab* **16**: 83–146

Duguet A, Bachmann P, Lallemand Y, Blanc-Vincent MP (2000) Bonnes pratiques diététiques en cancérologie: dénutrition et évaluation nutritionnelle [online], Fédération Nationale des Centres de Lutte Contre le Cancer (ed) November 2000. Available: URL: http://www.fnclcc.fr/-ci/sor/pdf/dietetique_evaluation_nutritionnelle_integrale_0899.pdf

Duguet A, Bachmann P, Lallemand Y, Blanc-Vincent MP (1999) Bonnes pratiques diététiques en cancérologie: dénutrition et évaluation nutritionnelle. *Bull Cancer* **86**: 997–1016

#### Nutrition consultation

Champetier S, Bataillard A, Lallemand Y, Montane C (2000) Bonnes pratiques pour la prise en charge diététique en cancérologie: la consultation. *Bull Cancer* **87**: 917–926

Champetier S, Bataillard A, Lallemand Y, Montane C (2000) Bonnes pratiques pour la prise en charge diététique en cancérologie: la consultation [online], Fédération Nationale des Centres de Lutte Contre le Cancer (ed) December 2000. Available: URL: http://www.fnclcc.fr/-sci/sor/pdf/consultation_dietetique_integrale_0500.pdf

### Genetic counselling

Eisinger F, Thouvenin D, Bignon YJ, Cuisenier J, Feingold J, Hoerni B, Lasset C, Lyonnet D, Maraninchi D, Marty M, Mattel JF, Sobol H, Maugard-Louboutin C, Nogues C, Pujol H, Philip T (1998) Réflexions sur l'organisation des consultations d'oncogénétique (première étape vers la publication de bonnes pratiques cliniques) In *Recommandations pour la pratique clinique en cancérologie [CD-ROM]*, Fédération Nationale des Centres de Lutte Contre le Cancer (ed) 2nd edn. Paris: FNCLCC, John Libbey EUROTEXT. Standards, Options et Recommandations

Eisinger F, Thouvenin D, Bignon YJ, Cuisenier J, Feingold J, Hoerni B, Lasset C, Lyonnet D, Maraninchi D, Marty M, Mattei JF, Sobol H, Maugard-Louboutin C, Nogues C, Pujol H, Philip T (1995) Réflexions sur l'organisation des consultations d'oncogénétique (première étape vers la publication de bonnes pratiques cliniques). *Bull Cancer* **82**: 865–878

### Nursing care

*Nurse delivered chemotherapy*

Teller AM, Bonnet C, Chalençon J, Deprados M, Doutre-Roussel D, Ehrhart M, Farsi F, Guicherd MF, Mathos G, Maurice F, Schlupp C, Schverzenzer N, Talon A, Titeux A (1998) Standards, options et recommandations pour une bonne pratique des soins infirmiers en chimiothérapie anticancéreuse. In *Recommandations pour la pratique clinique en cancérologie [CD-ROM]*, Fédération Nationale des Centres de Lutte Contre le Cancer (ed) 2nd edn. Paris: FNCLCC, John Libbey EUROTEXT. Standards, Options et Recommandations

Teller AM, Bonnet C, Chalençon J, Deprados M, Doutre-Roussel D, Ehrhart M, Guicherd MF, Mathos G, Maurice F, Schlupp C, Schverzenzer N, Talon A, Titeux A (1995) *Standards, options et recommandation: Bonne pratique des soins infirmiers en chimiothérapie anticancéreuse*. Paris: Expansion Scientifique Française, FNCLCC

#### Paediatric nursing care

Thinlot C, Farsi F, Castaing M, Le Ménager M, Lerouge I, Vasselon S, eds (2001) SOR: bonnes pratiques des soins infirmiers en pédiatrie. *Bulletin Infirmier du Cancer* **1**(Suppl. au n°2)

Thinlot C, Farsi F, Castaing M, Le Ménager M, Lerouge I, Vasselon S (2001) Standards, Options et Recommandations: bonnes pratiques des soins infirmiers en pédiatrie [online], Fédération Nationale des Centres de Lutte Contre le Cancer (ed) October 2001. Available: URL: http://www.fnclcc.fr/-sci/sor/pdf/infirmiers_pediatrie_integrale_0600.pdf

### Physical and social rehabilitation

Daly-Schveitzer N, Dilhuydy JM, Azadian-Boulanger G, Blanc-Vincent MP, Brugère J, Chollet A, Couette J, Decuypère ML, Desclaux B, Dessena J, Devinat M, Dumortier A, Eyffred M, Faucher A, Ladaique P, Laplante P, Le Guen C, Machet C, May-Levin F, Minier JF, Monira MH, Monticelli J, Netter E, Pommier P, Romieu G, Switsers O (1998) ‘Prévoir demain’: guide pour la réinsertion des patients traités pour cancers. In: Fédération Nationale des Centres de Lutte Contre le Cancer (ed) *Recommandations pour la pratique clinique en cancérologie [CD-ROM]*, 2nd edn. Paris: FNCLCC, John Libbey EUROTEXT. Standards, Options et Recommandations

### Epidemiology

#### Epidemiology of childhood cancers

Benhamou E, Laplanche A, Philip T (1998) Standards, Options et Recommandations. Epidémiologie descriptive des cancers de l'enfant (zéro à 14 ans) en France. In Recommandations pour la pratique clinique en cancérologie [CD-ROM], Fédération Nationale des Centres de Lutte Contre le Cancer (ed) 2nd edn. Paris: FNCLCC, John Libbey EUROTEXT. Standards, Options et Recommandations

#### Epidemiology of cancer in the adult

Esteve J, Benhamou E, Hill C, Laplanche A, Philip T (1998) Standards, options et recommandations. Epidémiologie descriptive des cancers de l'adulte en France. In *Recommandations pour la pratique clinique en cancérologie [CD-ROM]*, Fédération Nationale des Centres de Lutte Contre le Cancer (ed) 2nd edn. Paris: FNCLCC, John Libbey EUROTEXT; Standards, Options et Recommandations

